# Screening of Red Sea- and Mediterranean Sea-derived Actinomycetes for Antimicrobial and Antitumor activities: LC-ESI-HRMS-based Metabolomics Study

**DOI:** 10.1186/s12934-025-02759-0

**Published:** 2025-06-18

**Authors:** Mahmoud A. Abdel-Razik, Ahmed F. Azmy, Tarek Dishisha, Ahmed O. El-Gendy, Adlin Afzan, Nurkhalida Kamal, Ahmed Tawfike, Mohamed Sebak

**Affiliations:** 1 Department of Pharmaceutical Microbiology and Immunology, Faculty of Pharmacy, Beni-Suef University, Beni-Suef, 62514 Egypt; 2 Phytochemistry Unit, Herbal Medicine Research Centre, Institute for Medical Research, National Institutes of Health, Ministry of Health Malaysia, Shah Alam, Selangor 40170 Malaysia; 3 Institute of Systems Biology, Universiti Kebangsaan Malaysia, UKM, Bangi, Selangor 43600 Malaysia; 4 Department of Pharmacognosy, Faculty of Pharmacy, Helwan University, Cairo, 11795 Egypt

**Keywords:** Antimicrobial, Cytotoxicity, Dereplication, LC-HRMS, Marine actinomycetes, Metabolomics, NMR

## Abstract

In reply to the critical requirement for new anti-infective and anticancer agents, this study explores aquatic actinomycetes as a favorable source of naturally occurring bioactive compounds. Using metabolomics and dereplication analysis, we investigated the chemical profiles of promising eight actinobacterial isolates out of seventy-eight actinomycetes isolated from the Red Sea and the Mediterranean Sea. The eight isolates (6, 10, 12, 35, 42, 43, 45, and 48) demonstrated cytotoxic activity against the breast adenocarcinoma (MCF-7) cell line and broad-spectrum antimicrobial activity against Gram-negative and Gram-positive microbes. Isolate 10 exhibited the broadest antibacterial activity, while isolates 42, 43, and 45 showed moderate to weak cytotoxic potential. Genetic identification via 16S rRNA sequencing classified seven isolates as *Streptomyces* sp. and one as *Brevibacterium* sp. Multivariate data analysis (MVDA) of the high-resolution mass spectrometry (HRMS) data identified isolate 10 as an outlier, rich in diverse secondary metabolites. Dereplication revealed numerous metabolites with potential antimicrobial and anticancer activity, confirmed by NMR data highlighting their complex chemical profiles. These findings underscore the effectiveness of integrating metabolomics and bioassays to uncover unique bioactive products from aquatic actinobacteria, creating a foundation for further development of novel antimicrobial and antitumor medicines.

**Supplementary Information:**

The online version contains supplementary material available at 10.1186/s12934-025-02759-0.

## Introduction

Natural products are still employed to find pharmacologically active compounds, especially for the management of several diseases and cancers [1]. Between 1981 and 2014, more than 40% of anti-infective and anticancer drugs were developed from natural chemicals or semi-synthetic derivatives [2]. Nonetheless, more records of bacterial resistant to antibiotics are emerging in both hospital and community environments, posing the most significant challenge to researchers seeking to discover novel antimicrobial medicines [3]. As a result, growing interest is emerging in the development of novel naturally occurring therapies to fight bacterial resistant to antibiotics [4].

It is worth noting that actinomycetes accounted for about half of all documented microbial-derived bioactive chemicals [5], whereas *Streptomyces* was shown to generate around 75% of secondary metabolites produced by actinobacteria [6]. According to reports, marine actinomycetes comprise a high number of distinct structures with powerful bioactivities, as well as numerous novel species [7]. Several new bioactive chemicals have been identified in marine actinomycetes including marinomycins from *Marinophilus* sp., salinosporamide-A from *Salinispora* sp [8]., rifamycin from *Micromonospora* sp., marinopyrroles from *Streptomyces* sp., and abyssomicin-C from *Verrucosispora* sp [9].

Before initiating the time-consuming purification processes, metabolomics has been developed as a platform that may be used to thoroughly evaluate complex crude extracts and identify marker metabolites that may be linked to distinct biological potentialities. Chemometric studies of liquid chromatography-electrospray ionization-high-resolution mass spectrometry (LC-ESI-HRMS) and nuclear magnetic resonance (NMR) data enabled the analysis of a large array of metabolites within a given crude extract and determine how they relate to the targeted pharmacological activity [10]. Dereplication is frequently used in the process of isolating novel compounds and rapidly identifying already known natural products derived from microbial sources. This approach relies on comparing the compounds with previously reported secondary metabolites stored in natural product libraries and databases. It is a common technique in many drug discovery programs, particularly those that utilize LC-HRMS data [11]. Therefore, metabolomics and dereplication integrated approach minimizes redundancy, conserves time and resources and enhances the chances of discovering novel bioactive metabolites.

Despite its transformative potential, metabolomics- and bioassay-guided approaches have been underutilized in Egypt for investigating the chemical profiles of marine actinomycetes. Consequently, the aim of this study was to employ a metabolomics-guided plan, supported by bioassays and dereplication, to uncover novel bioactive secondary metabolites from marine actinomycetes. This integrated approach offers an opportunity to identify and target promising metabolites for further isolation and detailed chemical characterization.

## Materials and methods

### Samples collection

**Fig. 1:**
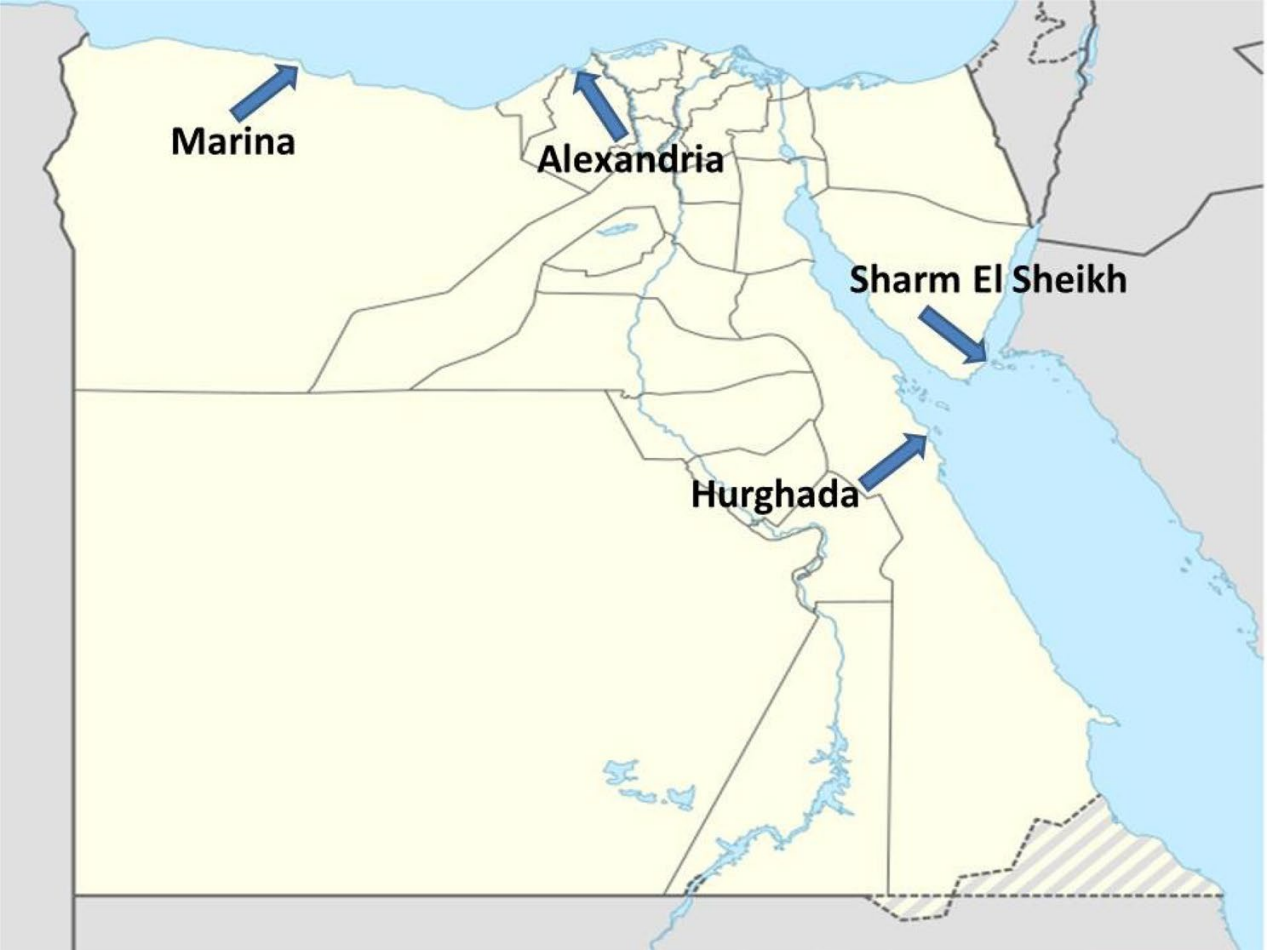
A map showing the sampling sites used in the present study. The four sampling sites used in the present study were Marina, Alexandria, Sharm El Sheikh, and Hurghada

In the current research, fourteen samples were obtained from marine water, sediment and plants between September and November 2020. The distribution and approximate geographic locations of the samples were as follows: (I) 5 samples (37%; 2 from sediment and 3 from water) from Hurghada, Red Sea Governorate, Egypt (27°15’26.57"N, 33°48’46.48” E); (II) 3 samples (21%; 1 from sediment, 1 from water, and 1 from plant) from Sharm El Sheikh, South Sinai Governorate, Egypt (27° 54’ 56.9412’’ N, 34° 19’ 47.8128’’ E); (III) 3 samples (21%; 1 from sediment, 1 from water and 1 from plant) from Alexandria, Alexandria Governorate, Egypt (31° 12’ 20.7108’’ N, 29° 55’ 28.2936’’ E); and (IV) 3 samples (21%; 1 from sediment, 1 from water and 1 from plant) from Marina, Marsa Matrouh Governorate, Egypt (30° 48’ 59.99” N, 29° 02’ 60.00” E) (Fig. 1). Using a hand-held soil borer, samples of sediments were collected from a depth of around 25 cm, while all water samples were taken at roughly 10 cm depth. Plants with different morphology were collected from mud and water at sampling locations. All samples were collected using 50 mL sterilized plastic containers with screw-cap lids and kept at -20 °C.

### Actinomycetes isolation

The marine plant samples were thoroughly cleaned with water for 3 to 5 min to eliminate organic and soil matter. They were then chopped into pieces of 1 cm^2^ and put into sterile conical flasks. Next, the surface of plant pieces were sterilized by submerging in ethanol (70%) for 3 min, NaOCl (0.4%) for 1 min, and ethanol (70%) for 2 min, succeeded by 3 rinses in distilled water for 1 min each time. Inside a laminar airflow chamber, samples were dried and the plant pieces were macerated with distilled water using a mortar and pestle before being cultured on different media listed below under the same conditions [12]. Also, the tissues that were sterilized on the surface were placed on the International *Streptomyces* Project-2 (ISP-2) agar medium and incubated at 30 °C to verify the effectiveness of surface sterilization [13].

The sediment samples were pre-treated by preserving them for 1 h at 50 °C in a water bath to aid the selection of actinomycetes [7]. Nine millilitres of sterile saline (0.9% NaCl) were used to dilute 1 mL of marine water or suspend 1 g of marine sediment, followed by dilution up to 10^− 5^, and homogenized by shaking on a rotary shaker at 150 rpm. An aliquot of 1 mL of the homogenate sample was inoculated onto the different media by spreading method and incubated aerobically at 30 °C for 7 to 20 days [7, 14, 15]. All samples (water, sediment, and plants) were inoculated on Starch Casein Agar (SCA), Kuster^ʼ^s agar (KA) and ISP-2 agar. To prevent fungal and Gram-negative bacterial contamination, each medium was treated with 50 µg/mL nystatin and 5 µg/mL rifampicin, respectively. Then, the colonies having actinomycete appearance were further purified [16].

### Screening for antimicrobial activity

The preliminary antimicrobial activity of our actinomycetes was assessed against several reference strains, including the Gram-positive bacteria *Listeria monocytogenes* (ATCC 7644) and methicillin-resistant *Staphylococcus aureus* (MRSA) (ATCC 43300), the Gram-negative pathogens, *Salmonella enterica* (ATCC 14028) and *Escherichia coli* (*E. coli*) (ATCC 25922), and the yeast-like *Candida albicans* (ATCC 60193) by the agar diffusion method [17]. Briefly, a pure colony of test bacteria was placed in sterile normal saline until the visual turbidity reached 0.5 McFarland. A swab was immersed in bacterial solution and distributed above nutrient agar medium. Each actinomycete was grown on ISP-2 agar and *Streptomyces* Antibiotic Activity (SAA) agar for 7 days at 30 °C. Then, agar discs (7 mm) were cut from each actinomycete’s ISP-2 and SAA agar media and transferred to pre-inoculated nutrient agar. Next, the plates were subjected to pre-diffusion at 4 °C for 90 min, and after incubation for 24 h, the antimicrobial activity findings were evaluated. Finally, zones of inhibition equal to or larger than 12 mm were a sign of positive antibacterial or antifungal activity of our actinomycetes [17].

The secondary antimicrobial activity screening of the most active isolates was made against some drug-resistant strains (clinical isolates recovered from a microbiological laboratory in Beni-Suef city, Egypt), including the Gram-positive bacteria *Enterococcus* sp. and *Staphylococcus aureus* 2, and the Gram-negative bacteria *Pseudomonas* sp., *Klebsiella* sp., and *Enterobacter* sp. by the agar diffusion method described above [17].

### Molecular characterization of selected isolates

The genomic DNA of the chosen bioactive isolates (8 isolates) was extracted using Quick-DNA™ Fungal/Bacterial Miniprep Kit (Zymo Research, USA) as instructed by the manufacturer. A polymerase chain reaction (PCR) targeting the 16S rDNA gene was then conducted on the extracted DNA using the forward primer 27 F (5’-AGAGTTTGATCITGGCTCAG-3’) and the reverse primer 1492R (5’-ACGGITACCTTGTTACGACTT-3’). The total PCR reaction was 50 µL comprising 25 µL COSMO PCR RED Master Mix (Willowfort, UK), 2 µL of each primer, 1 µL of extracted DNA, and nuclease-free water to 50 µL. The PCR amplification was run as follows: initial denaturation (95 °C) for 2 min, succeeded by 35 cycles of denaturation (95 °C) for 15 s, annealing for 20 s at 65 °C, and extension at 72 °C for 30 s, with final extension (72 °C) for 10 min. After purification of the PCR product using DNA clean and Concentrator − 25 kit (Zymo Research, USA), the purified PCR products were sent to GACT (Germany) for sequencing. The National Center for Biotechnology Information (NCBI) nucleotide basic local alignment search tool (BLASTn) was used to get the closest sequences from the GenBank database using the obtained 16S rDNA gene sequences as a query (https://blast.ncbi.nlm.nih.gov/Blast.cgi?PAGE%20TYPE=BlastSearch). The best-matched sequences were subsequently aligned using the MUSCLE tool [18], which was included in the MEGA 6.0 software package [19]. A phylogenetic tree depicting the evolutionary connections between the aligned sequences was then created using the neighbor-joining method [20] and Kimura-2-parameter model distances [21], integrated within the above-mentioned software, with bootstrapping (1,000 iterations). The dataset was cleaned by removing all gaps and missing data points using the “complete deletion” option.

### Fermentation and extraction of the secondary metabolites

Biological active selected isolates were cultivated on SAA agar for 7 days at 30 °C. A pure colony from plates was then inoculated into 200 mL ISP-2 broth in 1 L Erlenmeyer flasks and the flasks were incubated on a rotatory shaker at 30 °C for 5 days at 150 rpm. The bacterial broth was filtered. The filtration was used as the actinomycete pellets are large enough to be filtered and because filtration requires less energy, easier to be accomplished and cost-effective, while filtration was suggested in several studies [17, 22]. Also, filtration does not require rapid spinning, thereby avoiding the heat and shear forces that could damage delicate natural compounds. Then 1:1 *v/v* of freshly ethyl acetate was added to the bacterial broth to inhibit the microbial growth and the mixture was vigorously shaken before being allowed to sit overnight. To extract the maximum amount of actinobacterial natural compounds, liquid-liquid extraction was conducted once between broth and freshly ethyl acetate (1:1 *v/v*). The extracts of ethyl acetate were finally evaporated, and the dry microbial extracts were stored for future use [23].

### In vitro anti-tumor cytotoxicity

The Sulforhodamine B (SRB) test was employed to assess the cytotoxicity of the selected bioactive actinomycetes, as previously described [24, 25] with few modifications. Briefly, the chosen cancer cell lines were breast adenocarcinoma, MCF-7, colorectal cancer cells, HCT-116, and the normal cell line, human skin fibroblasts (HSF). Briefly, the HSF and MCF-7 cell lines were preserved in Dulbecco’s Modified Eagle Medium (DMEM), while the HCT-116 cell line was preserved at Roswell Park Memorial Institute (RPMI) medium. In a humidified environment with 5% (*v/v*) CO_2_ at 37 °C, the two mediums were augmented with heat-inactivated fetal bovine serum (10%), streptomycin (100 units/mL), and penicillin (100 units/mL). To enable the attachment of cells to the plate wall, portions of 100 µL cell suspension (5 × 10^3^ cells) were placed in 96-well plates and incubated in full medium for a full day. The cells were treated with another aliquot of 100 µL media including serial concentrations of standard anticancer drug (5-fluorouracil (5-FU)) and various extracts in concentrations of 128, 32, 8, 2, 0.5 µg/mL. The growth of the cell lines was monitored using untreated cells as a control and culture media (DMEM and RPMI) as a blank. After the cells were treated with the extracts and standard drug for 72 h, they were fixed by adding 150 µL of 10% trichloroacetic acid (TCA) to the medium and incubating for 1 h at 4 °C. After the TCA solution was removed, the cells were washed 5 times with distilled water. Aliquots of 70 µL SRB solution (0.4% w/v) were incubated for 10 min at room temperature in a dark area. Next, 3 times 1% acetic acid washes were done, and the plates were left to air dry for the whole night. Finally, 150 µL of Tris(hydroxymethyl)aminomethane (TRIS) (10 mM) was added to dissolve the protein-bound SRB stain, and a BMG LABTECH^®^-FLUOstar Omega microtiter plate reader (Ortenberg, Germany) was used to detect the absorbance at 540 nm. For each tumor cell line, the relationship between extract concentrations and viable cells was plotted to create a survival curve, and the IC_50_ was also computed. The standard antitumor drug (5-FU) was used in the experiment, which was conducted in triplicates.

The current study’s cytotoxicity data were statistically examined using Excel. After calculating and expressing the mean ± SD of the triplicate tests, all IC_50_ values were assessed. Using an unpaired student’s t-test, the IC_50_ values of the bacterial extracts against the tested tumor cell lines and the standard drug, 5-FU, were compared with those against the normal cell lines to determine the statistical significance. Statistics were deemed statistically significant when *p* < 0.05.

### NMR spectroscopy

Biologically active selected isolates were cultivated on SAA agar for 7 days at 30°C. A pure colony from plates was then inoculated into 200 mL ISP-2 broth in 1 L Erlenmeyer flasks and the flasks were incubated on a rotatory shaker at 30°C for 5 days at 150 rpm. The bacterial broth was filtered, and then the liquid-liquid extraction was conducted twice between broth and ethyl acetate (1:1 *v/v*) as mentioned above. The extracts of ethyl acetate were finally evaporated, and the dry microbial extracts were used. Eight milligrams of selected actinobacterial extracts and blank medium were dissolved in 200 µL DMSO-d_6_ and transferred to NMR tubes (3 mm in diameter and 7” length) to run proton NMR (^1^H NMR) [26] using Avance III 400 FT-NMR spectrometer with magnet Ascend™ 400 MHz UltraShield™ (Bruker, Switzerland). The ^1^H NMR experiments were run at 16 scans. MestReNova (Mnova 10.00, Mestrelab Research SL, US) software was used to process the proton NMR spectra as previously described [22]. The ^1^H NMR experiment was run in triplicates.

### LC-MS/MS acquisition method

The untargeted LC-MS/MS analyses of the crude actinobacterial extracts and blank medium were conducted on a Q-Exactive Mass Spectrometer equipped with an electrospray interface (Thermo Fisher Scientific, Massachusetts, USA). An ACQUITY UPLC^®^ HSS T3 column, measuring 100Å, 1.8 μm, 2.1 × 100 mm (Waters^®^, Massachusetts, USA) was used for the chromatography and the column was preserved at 40 °C throughout the analysis. The samples were prepared at 5 mg/mL concentrations in methanol. To check for system stability, quality control (QC) samples were analyzed six times at the beginning of the sequence and after every five samples. QC samples were prepared by mixing equal parts of each sample. Additionally, all samples were analyzed in randomized order to minimize systematic bias. The mobile phases comprised water (solvent A) and acetonitrile (solvent B), each containing 0.1% formic acid (v/v). All mobile phases were LC-MS grade solvents (Thermo Fisher Scientific, Massachusetts, USA). A 15-minute elution method was delivered at a flow rate of 0.3 mL/min: 10% B isocratic from 0 to 1 min, 10–30% B from 1 to 8 min, 30–100% B from 8 to 10 min, 100% B isocratic from 10 to 12 min, ramp to 10% B from 12 to 12.5 min and a column equilibration step with 10% B from 12.5 to 15 min. The autosampler was kept at 4 °C during analysis and the injection volume was set to 1 µL.

Samples were analyzed in both positive (PI) and negative ionization (NI) modes. The ion source parameters were spray voltage of 4.0 kV (positive), 3.2 kV (negative), capillary temperature of 350 °C, sheath gas pressure of 30 psi, auxiliary gas pressure of 15, maximum spray current of 100, probe heater temperature of 60 °C, and S-Lens RF level of 55%. The data-dependent MS/MS method included a full scan MS (*m/z* 100–1500) operated at 70,000 resolutions and 250 ms maximum IT. This was followed by an MS/MS scan of the top five most abundant ions at 17,500 resolutions and 60 ms maximum IT. Parent ions were placed in a dynamic exclusion list for 3.0 s and the MS/MS isolation window width was 4 *m/z*, while the isotopes were excluded. The LC-MS experiment was run in triplicates.

### Metabolomics data analysis and dereplication study

The raw HRMS/MS data in both PI and NI ionization modes were converted to mzML format using the ProteoWizard MassConvert program [27] and these files were processed separately in MZmine 2.53 to extract features from the raw data [28],. Data processing was performed for each data set using previously described multi-step approach [29].

For additional data cleaning, the CSV files produced from the data processing steps were transferred into an in-house Excel macro [26]. In summary, background ion peaks in the solvent blank and medium were subtracted. Then, to prepare for multivariate data analysis, the processed data files for the PI and NI modes were combined simultaneously. Lastly, the metabolites in the samples were dereplicated to those compound lists in the Dictionary of Natural Products (DNP) database. Every solvent blank peak with a peak intensity higher than 1 × 10^4^ was manually removed from the sample’s peak list. Using an algorithm that determines each *m/z* intensity in both the medium and bacterial extracts, peaks from the ISP2 media were also deducted from the samples. Only features with peak intensities 20 times higher in the samples than in the ISP2 medium remained in the final peak list. Finally, each *m/z* ion peak was dereplicated against the customized database (DNP) using the Excel macro using a mass error of 5 ppm.

To prepare for the MVDA, the Excel macro-generated SIMCA data sheet was exported to SIMCA 14.0 software (Umetrics, UmeŇ, Sweden). For unsupervised MVDA, Principal Component Analysis (PCA) was constructed, while for supervised analysis, Partial Least Squares Discriminant Analysis (PLS-DA) and Orthogonal Partial Least Squares Discriminant Analysis (OPLS-DA) were used to evaluate the data set. All models were built based on Pareto scaling. OPLS-DA was applied to compare the chemical profiles of the isolates after pre-grouping them based on their bioassay screening results, while PCA was engaged to analyze and compare the chemical profiles of the samples without any prior classification. Pearson correlation of significantly different metabolites was created using Graph Pad Prism 9.0, whereas Random Forest analysis was carried out using Metaboanalyst 6.0. A heat map was created using the LC-HRMS data of all bioactive isolates to demonstrate the intensity of key chemicals in the chemical profiles of our extracts and to correlate the bioactivity of the extracts with their chemical profiles.

By choosing the discriminatory molecules present in the biological active extracts and contrasting them with the dereplication data, the metabolomics profile tables of remarkable biological active metabolites were inferred from the S-plots of the OPLS-DA. The interesting actinobacterial hits were further cross checked using the online natural product database that can be accessed for free at http://www.knapsackfamily.com/knapsack_core/top.php. Based on the outcomes of the dereplication study, bioassay analysis, and MVDA of the biological active extracts, favorable isolates were identified.

Finally, the MVDA of the proton NMR spectra was performed using SIMCA 14 after data processing of NMR data using MestReNova 10.00 followed by deleting water and DMSO peaks in Microsoft EXCEL [22].

## Results and discussion

### Isolation of actinomycetes

**Fig. 2:**
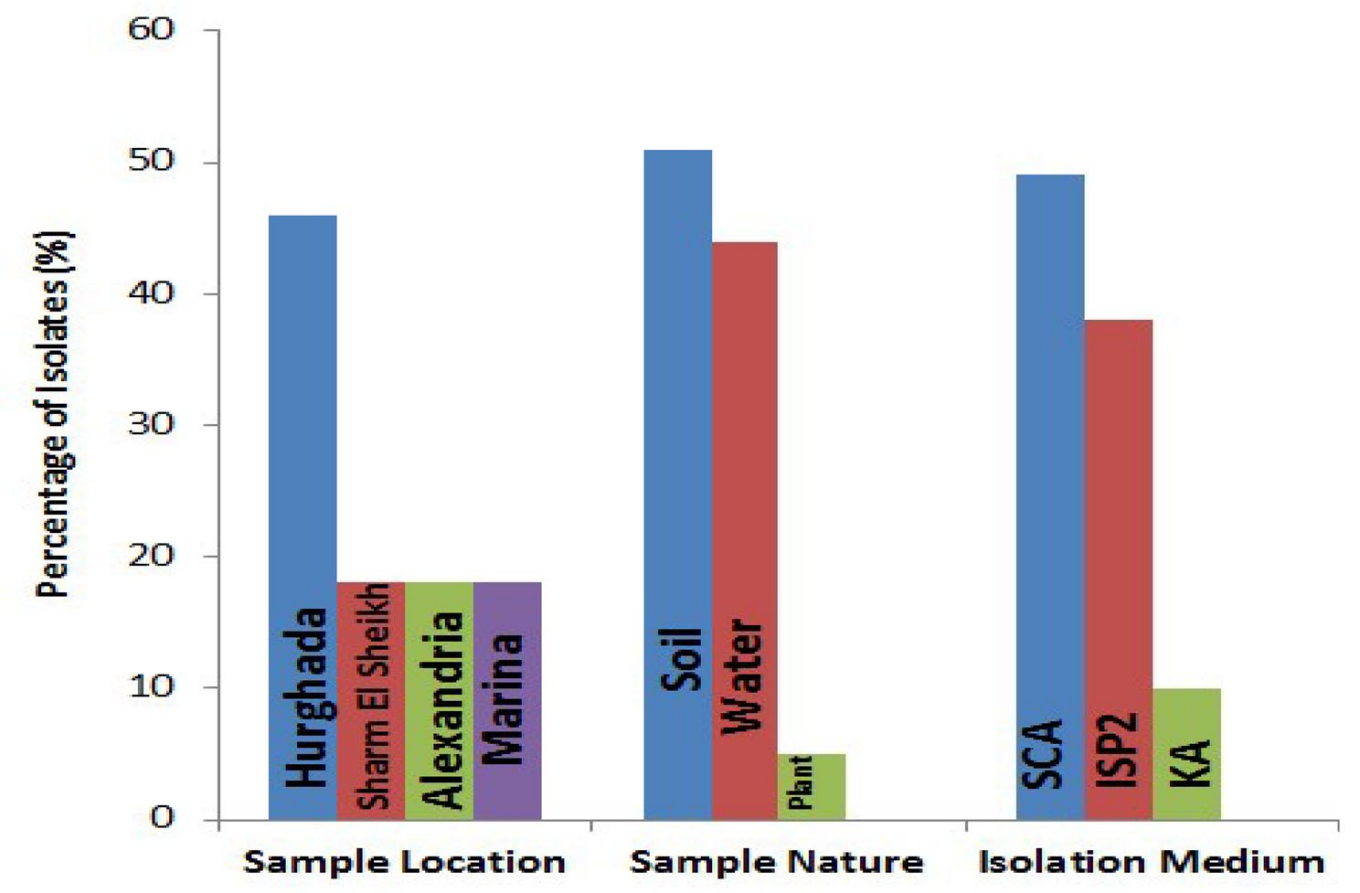
Distribution of actinobacterial isolates according to sample location, sample nature, and isolation media

In this study, seventy-eight bacterial isolates were recovered from sediment, water and plant samples that have been collected from several marine environments in Egypt. Based on their microscopical inspection, mycelium discoloration, pigment diffusion, and colonial shape, the isolates were phenotypically considered to be actinomycetes. This included 40 isolates (51%) from sediment samples, 34 isolates (44%) from water samples and 4 isolates (5%) from plant samples (endophytes) (Fig. 2). Many earlier findings verified the abundance and essential role of significant actinomycetes in many ecosystems including marine environments [30]. Actinomycetes are found scattered across the strata of marine sediments, where they aid in the breakdown of carbon-based materials and nutrients turnover. These microbes can float or adhere to particle debris in water bodies, which can affect the dynamics of the ecosystem and the makeup of the microbial community. To support plant development and defense systems, actinomycetes frequently establish symbiotic interactions with marine plants. Their prevalence on plants, water, and sediment emphasizes their importance in marine ecosystems as well as their potential for use in biotechnology [31].

Notably, a total of 38 isolates (49%), 30 isolates (38%), and 10 isolates (13%) were recovered using SCA medium, ISP-2 agar medium and KA medium, respectively (Fig. 2). These media are commonly used by many researchers to isolate marine actinomycetes [12]. Marine actinomycetes’ isolation and culture on various growth media are essential for researching their variety and their uses. A popular medium for isolating marine actinomycetes, the SCA medium offers a wealth of nutrients that promote the development of these filamentous bacteria. Actinomycetes are encouraged to develop and sporulate by the presence of casein and starch in this media, which act as sources of nitrogen and carbon, respectively. ISP2 medium contains dextrose, yeast extract, and malt extract, these standard carbon and nitrogen sources can support the growth of a wide range of bacteria including actinomycetes strains. Glycerol, casein, and other salts are frequently present in the KA medium, which gives actinomycetes vital nutrients and promotes their growth. However, in comparison to other media, such as SCA and ISP2, several research studies revealed low growth in KA. These varied media give scientists useful instruments for separating and examining the traits of actinomycetes in different settings, which advances our knowledge of their ecological functions and biotechnology possibilities [32, 33].

Regarding the distribution of isolates by sampling locations, 36 isolates (46%) were recovered from Hurghada, 14 isolates (18%) were isolated from Sharm El Sheikh, and 14 isolates (18%) were obtained from Alexandria while the remaining 14 isolates (18%) were attained from Marina in Egypt (Fig. 2). The total number of isolates recovered from the Red Sea (Hurghada and Sharm El Sheikh) is 50 isolates (64%), while 28 isolates (36%) were recovered from the Mediterranean Sea (Alexandria and Marina). Actinomycetes were previously found to be widespread in the Mediterranean Sea and the Red Sea in Egypt. They form a distinct marine ecosystem characterized by elevated salinity, high water temperatures and a rich diversity of microbial life, setting them apart from other tropical seas [34, 35].

### Evaluation of the antimicrobial activity

The preliminary screening for antimicrobial activity of our actinobacterial isolates against 5 reference strains showed that 59 isolates (76%) have positive antimicrobial activity against a minimum of one indicator microorganism. Out of bioactive isolates, 49 (83%) isolates were active against MRSA, 45 (76%) were active against *E. coli* and *Salmonella enterica*, 42 (71%) were active against *Listeria monocytogenes*, and 39 (66%) were active against *Candida albicans* (Table 1). The differences in sensitivity between Gram-negative and Gram-positive bacteria towards the antimicrobial compounds secreted by the actinomycetes can be attributed to the combined effects of their cell wall structure, outer membrane barriers, genetic resistance mechanisms and nature of antimicrobial compounds [36]. As highlighted in numerous earlier reports [33, 37, 38], the marine ecosystem is regarded as an abundant source of actinomycetes with antimicrobial properties. A previous study [39] illustrated that 12% of marine actinomycete isolates had activity against *Staphylococcus aureus* (ATCC6538) and *E. coli*, while 30% of isolates were active against *Pseudomonas aeruginosa* (ATCC 6739).

**Table 1:** Result of antimicrobial activity of selected actinomycetes against different reference strains and MDR isolates using agar diffusion method. Positive antimicrobial activity was recorded for any inhibitory zone with a diameter equal to or more than 12 mm

Isolate No.	Zone of inhibition (mm)									
	MRSA	Listeria monocytogenes	Candida albicans	Salmonella enterica	E. coli	Enterobacter sp.	Pseudomonas sp.	Klebsiella sp.	Enterococcus sp.	Staphylococcus aureus 2
6	23	22	20	22	26	18	NA	25	13	21
10	23	23	22	21	24	24	13	26	16	25
12	22	22	19	23	21	20	NA	26	15	23
35	30	27	17	27	26	23	NA	25	13	26
42	29	25	16	23	21	21	NA	24	15	25
43	32	28	24	27	27	23	NA	27	13	25
45	30	28	20	25	27	22	14	26	NA	27
48	31	35	20	25	28	15	NA	20	14	25

Moreover, our findings showed that 26 isolates (33.33%) were active against all tested reference strains in the preliminary screening of the antimicrobial activity. These twenty-six isolates were chosen for secondary analysis of their antimicrobial potentiality against some multi-drug-resistant (MDR) clinical strains. A serious and growing hazard to public health across the world is bacterial resistance, which is a consequence of excessive use and abuse of antibiotics. The treatment of bacterial infections is significantly hampered by bacteria’s capacity to acquire resistance genes or create resistance mechanisms against antibiotics through genetic alterations by mutations or horizontal gene transfer. Mutations generate new genetic variations at a slow pace, but they can directly cause resistance when they alter antibiotic targets or regulatory genes. Horizontal gene transfer (HGT) processes-such as transformation, transduction and conjugation-facilitate the swift uptake and spread of resistance genes, frequently enabling bacteria to rapidly adapt to antibiotics and various environmental challenges [40]. This issue hinders the finding of novel medications besides decreasing the efficacy of currently available antibiotics. The rise of MDR bacteria, sometimes known as “superbugs”, has resulted in more treatment failures, longer hospital stays, increased medical expenses, and greater death rates [41].

Surprisingly, we found that all 26 tested actinomycetes were active against *Klebsiella* sp. and *Staphylococcus aureus* 2, 22 isolates (85%) were active against *Enterobacter* sp., 7 isolates (27%) were active against *Enterococcus* sp. and 2 isolates (8%) were active against *Pseudomonas* sp. (Table 1). As shown in a previous study [32], 15% of marine actinomycete isolates had antibacterial activity against MDR bacteria, especially methicillin-resistant *Staphylococcus aureus* and vancomycin-resistant *Enterococcus* (VRE).

**Fig. 3:**
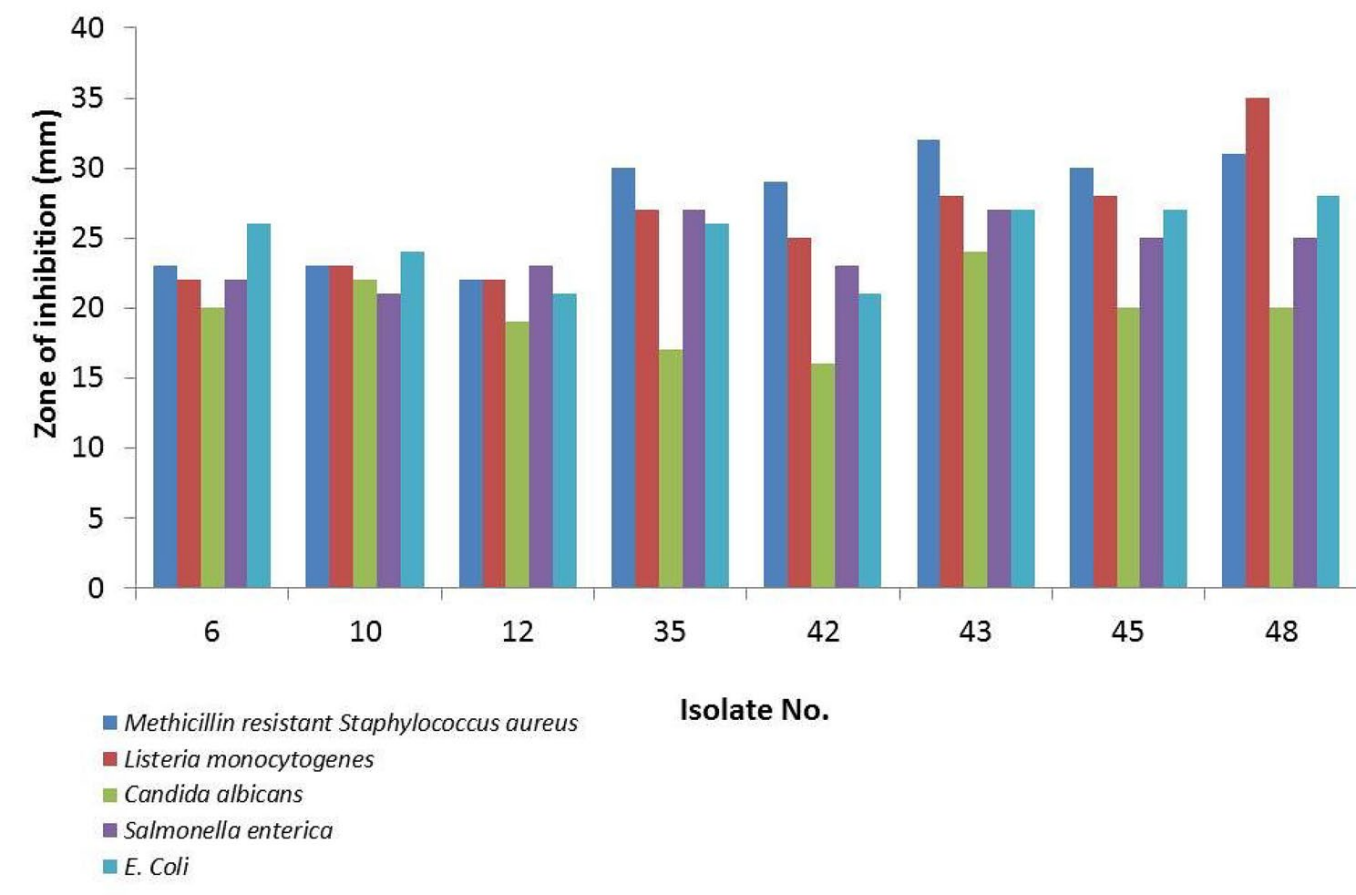
Antimicrobial activity result expressed as zones of inhibition of the selected bioactive actinomycetes against reference strains

**Fig. 4:**
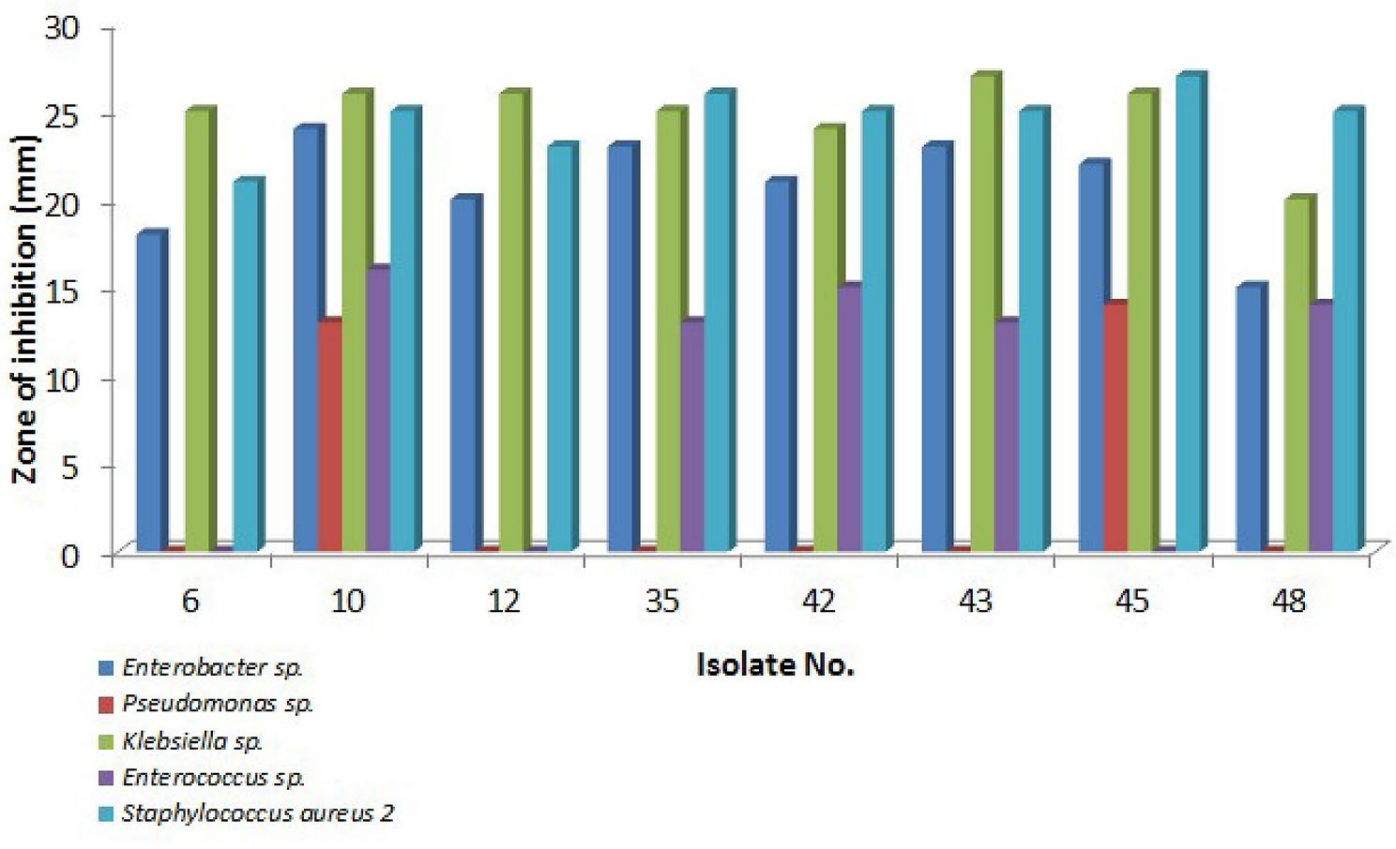
Antimicrobial activity result expressed as zones of inhibition of the selected bioactive actinomycetes against the MDR clinical strains

Although the majority of the tested isolates demonstrated activity against a minimum of one test microorganism, only eight bioactive isolates showed activity against four multi-drug-resistant isolates at least (Figs. 3 and 4) and therefore they were selected for further analyses.

### Molecular characterization

**Fig. 5:**
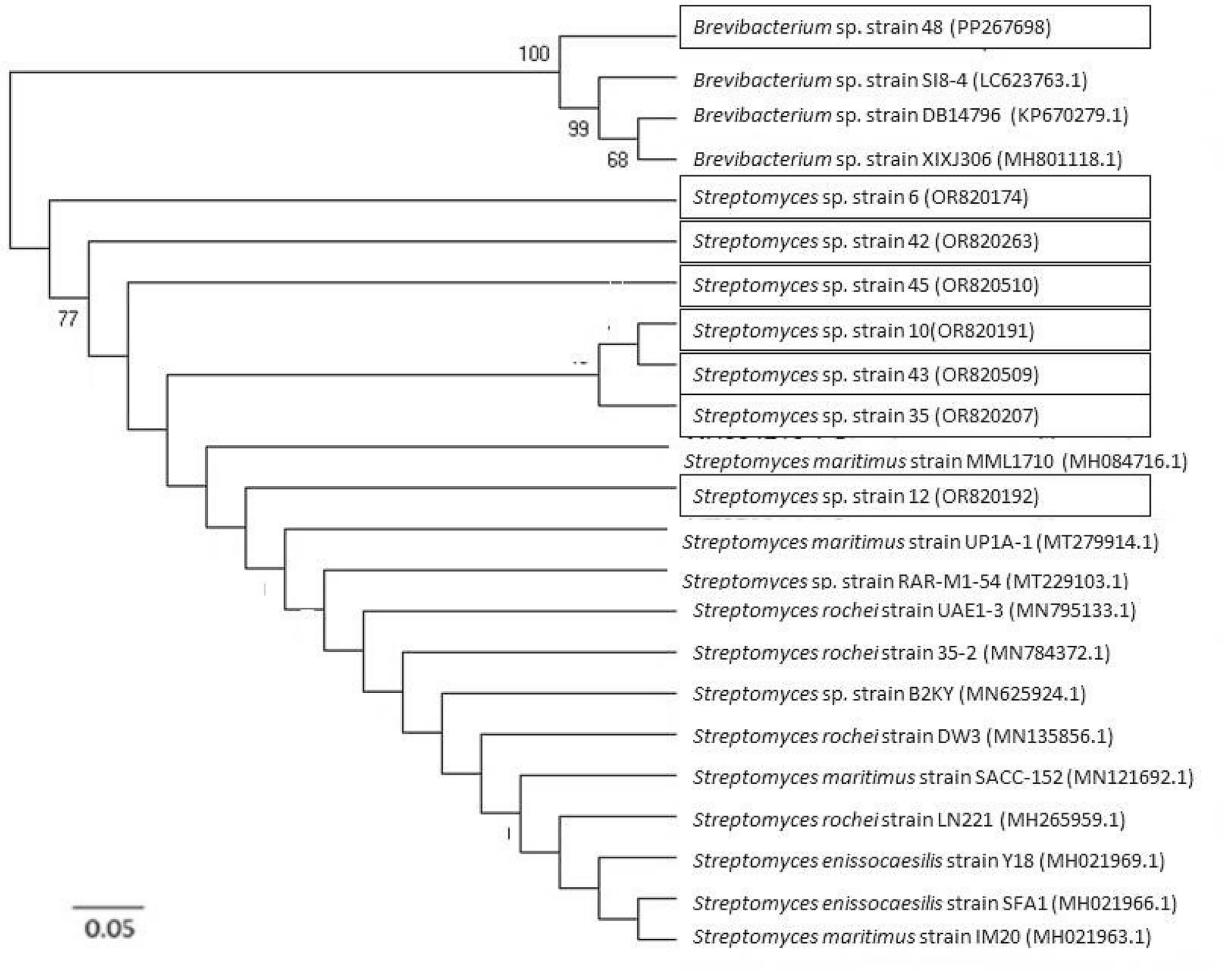
Phylogenetic tree of isolates based on 16S rRNA gene sequence analysis using neighbor joining method and kimura-2-parameter model distances available within MEGA7 software. Bootstrap values, shown at the nodes, were calculated from 1000 replicates. Bootstrap values below 60% are not displayed. The scale bar indicates substitutions per nucleotide. The GenBank accession numbers for the 16S rDNA sequences are given in parentheses after the strain. The tree was rooted

The homology search for sequences of the 16S rRNA gene for the eight chosen actinobacterial isolates using the MegaBLAST tool in NCBI revealed that seven sequences had close similarity (more than 99%) with various *Streptomyces* species and only one sequence displayed very high similarity (more than 97%) with various *Brevibacterium* species in GenBank (Table 2). Multiple sequence alignments were done on our sequences with different 16S rRNA gene sequences from NCBI, and a phylogenetic tree was created (Fig. 5). Therefore, seven isolates (6, 10, 12, 35, 42, 43 and 45) were recognized as *Streptomyces* sp. whereas, only one isolate (48) was recognized as *Brevibacterium* sp. based on their similarity to multiple sequences of various *Streptomyces* and *Brevibacterium*, respectively. These findings are strongly supported by prior results from several research studies that the genus of *Streptomyces* and *Brevibacterium* were widespread in marine environments [42, 43].

### In vitro antitumor cytotoxicity

**Fig. 6:**
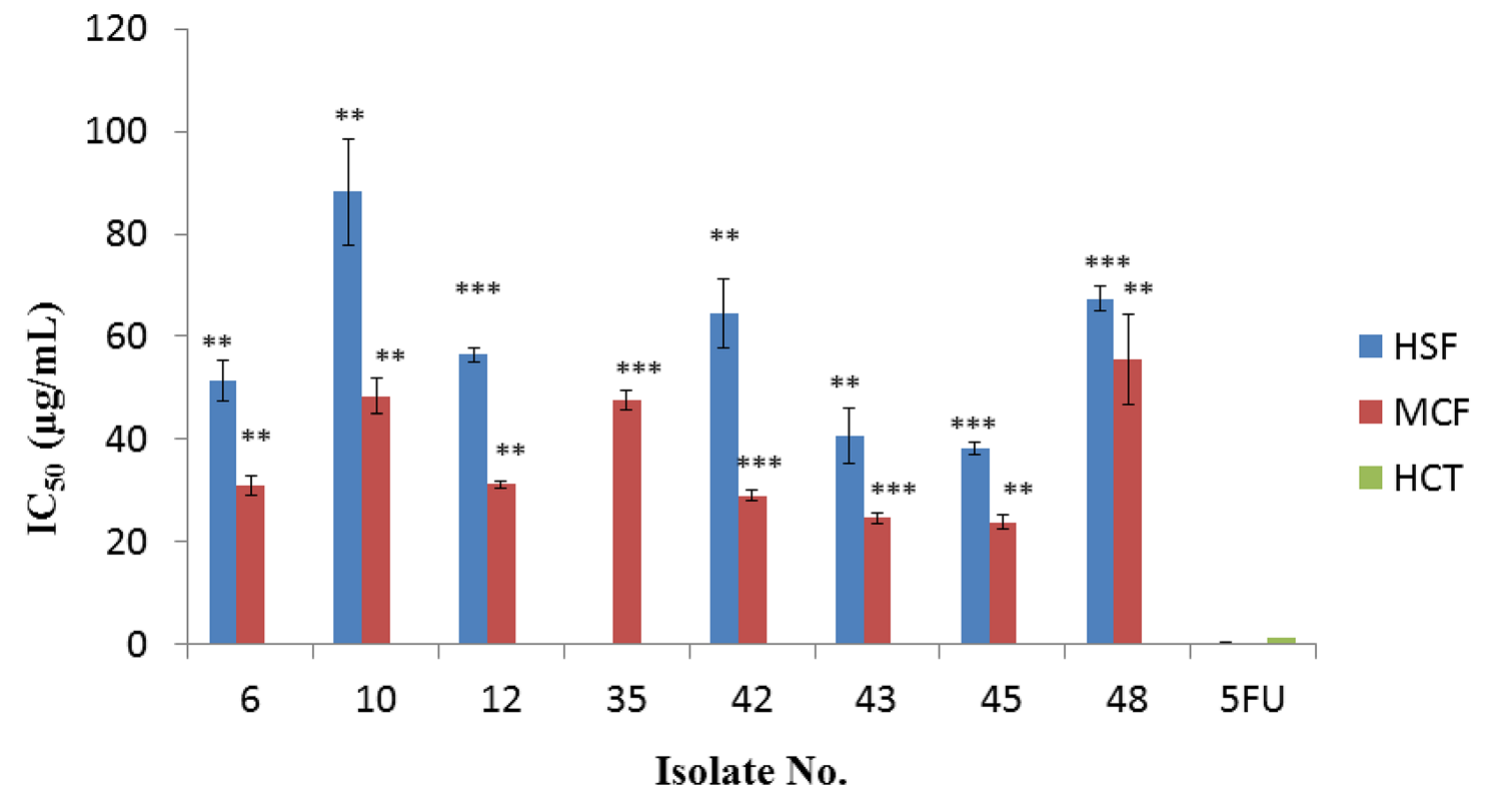
Cytotoxic activity of the selected bioactive isolates against the cancer cell lines (HCT-116 and MCF-7) and normal cell lines (HSF). Values represent the IC_50_ (µg/mL) expressed as the mean ± the SD of three independent replicates. A paired two-tailed distribution Student t-test was conducted to compare the significance between mean values, with 5-FU as a reference (*, *p* > 0.05; ** *p* > 0.01; ***, *p* > 0.001)

Researchers are still looking for natural sources of anticancer chemicals, which have aided in the evolution of new medications for malignancy therapy [44]. All eight isolates that showed potential antimicrobial activity were assessed for their cytotoxic activity on two cancer cell lines, MCF-7 and HCT-116, in addition to the normal cell line, HSF. All investigated isolates showed moderate to weak antitumor activity against MCF-7 but no inhibitory efficacy against HCT-116 (Table 3). The isolates 45, 43 and 42 showed moderate to weak anticancer potential against MCF-7, with IC_50_ values of 23 ± 1.45, 24 ± 0.93 and 29 ± 1.04 µg/mL, respectively (Fig. 6). The findings of the cytotoxicity assay for our extracts were compatible with various earlier studies [44, 45]. For example, two extracts from different marine sediment actinomycetes (*Streptomyces* sp. ACT01 and ACT02) had cytotoxic activity against the MCF-7 cell line with IC_50_ values of 10.13 ± 0.92 and 22.34 ± 5.82 µg/mL, respectively [45]. Also, crude extract of marine actinobacterium *Streptomyces parvus* displayed cytotoxic activity against MCF-7 with 57% inhibition activity [39]. On the other hand, several compounds isolated from actinomycetes have exhibited cytotoxic potentialities against several cell lines. For instance, *Streptomyces* sp. KML-2 produced antitumor compounds such as 1-(1 H-indol-3-yl)-propane-1,2,3-triol and chromomycin SA with IC_50_ values of 0.97 and 12.6 *µ*g/mL against MCF-7 and, 7.8 and 8.9 *µ*g/mL against HeLa (cervical cancer cell line), respectively [46]. In addition, benzoxazole derived from soil *Streptomyces* sp. Tü 6176 showed strong antitumor activity against AGS (gastric adenocarcinoma), MCF-7 and HepG2 (hepatoblastoma cancer cell line), with IC_50_ values of 0.4, 0.68 and 0.06 *µ*g/mL, respectively [47]. Moreover, di-(2-ethylhexyl) phthalate that was isolated from *Streptomyces mirabilis* NSQu-25 showed antitumor activity against MCF-7, HepG2, and HCT 116 with IC_50_ values of 6.941, 9.028 and 3.681 *µ*g/mL, respectively [48].

**Table 2:** Cytotoxic activity of the selected actinobacterial isolates against the cancer cell lines (HCT-116 and MCF-7) and the normal cell lines (HSF). 5-FU = 5-Fluoro uracil

Isolate No.	IC_50_ (µg/mL)		
	HCT-116	MCF-7	HSF
6	NA	30.91 ± 1.84	48.93 ± 3.95
10	NA	48.36 ± 3.42	88.16 ± 10.23
12	NA	31.17 ± 0.73	56.51 ± 1.39
35	NA	47.61 ± 1.84	NA
42	NA	29 ± 1.04	64.43 ± 6.86
43	NA	24.62 ± 0.93	40.74 ± 5.31
45	NA	23.78 ± 1.45	38.15 ± 1.18
48	NA	55.50 ± 8.91	67.37 ± 2.33
5-FU (Standard)	1.25 ± 0.18	0.011 ± 0.01	0.22 ± 0.04

**Table 3:** The similarity between each isolate and its closest species from NCBI database

Isolate No.	Closest Species (Accession Number)	Percent Identity
*Streptomyces* sp. strain 6	*Streptomyces maritimus* strain IM20 (MH021963)	more than 99
	*Streptomyces maritimus* strain TIA3 (MT954062)	
*Streptomyces* sp. strain 10	*Streptomyces rochei* strain APS29 (MH021962)	
	*Streptomyces rochei* strain JK1 (CP121271)	
*Streptomyces* sp. strain 12	*Streptomyces vinaceusdrappus* strain AC-40 (CP104697)	
	*Streptomyces maritimus* strain MML1710 (MH084716)	
*Streptomyces* sp. strain 35	*Streptomyces rochei* strain D21E05 (CP139824)	
	*Streptomyces rochei* strain SR1102 (CP161948)	
*Streptomyces* sp. strain 42	*Streptomyces rochei* strain NS1 (OL744624.1)	
	*Streptomyces enissocaesilis* strain Y18 (MH021969.1)	
*Streptomyces* sp. strain 43	*Streptomyces rochei* strain NBRC 12,908 (NR_041091.1)	
	*Streptomyces rochei* strain A-1 (GQ392058.1)	
*Streptomyces* sp. strain 45	*Streptomyces enissocaesilis* strain Man (OR447473.2)	
	*Streptomyces plicatus* strain NBRC 13,071 (NR_112357.1)	
*Brevibacterium* sp. strain 48	*Brevibacterium epidermidis* strain P159 (NR_029262.1)	more than 97
	*Brevibacterium linens* strain BS258 (CP014869.1)	

### Metabolomics data analysis and dereplication study

**Fig. 7:**
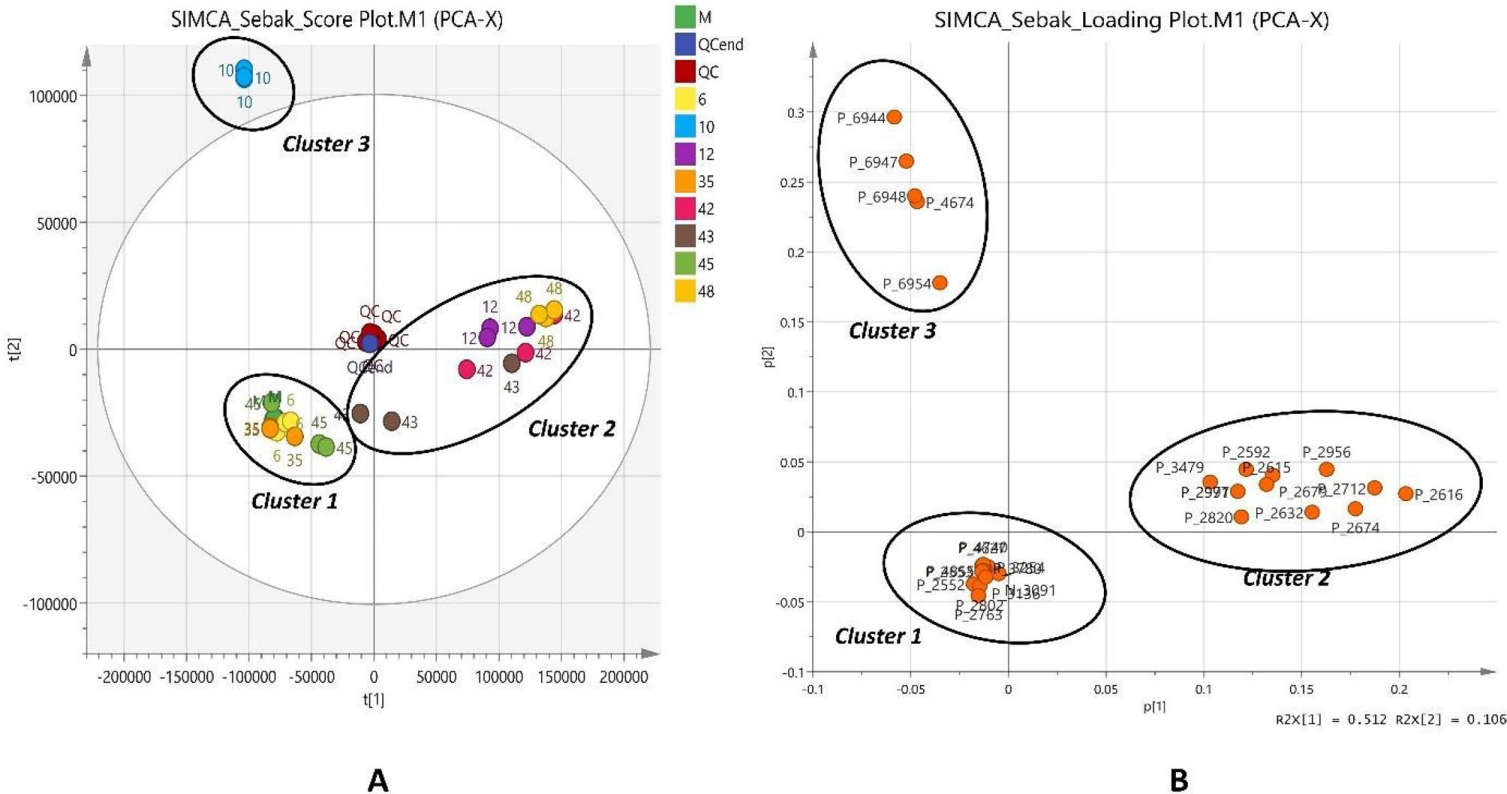
The multivariate variable pattern analysis for the normalized data was conducted using SIMCA software version 14.0. (**A**) Principal component analysis (PCA) score plot derived from the LC–HR-MS profiles of unlike sample groups: Media (M), quality control (QC), and various microbes’ isolation groups: 6, 10, 12, 35, 42, 43, 45, 48. (**B**) PCA loading plot displaying the clustering of metabolite content in unlike sample groups. Metabolites in 3 apparent clusters, Cluster 1, Cluster 2 and Cluster 3

The MVDA was performed to evaluate differences in metabolite contents across sample groups. To determine the metabolites accountable for the noted variations and to uncover clustering patterns within each sample group, PCA was utilized. Sample grouping is graphically demonstrated by the PCA score plot, and the variables causing these differences are explained by the loading plot. According to the PCA results (Fig. 7), PC1 and PC2 respectively described 51.2% and 10.6% of the variance in the data. The 95% confidence Hotelling T2 area is shown by the ellipse on the score plots, and all samples are shown to fall within the ellipse except for the sample group of isolate 10 which falls outside of the ellipse. The centre of the PCA plot showed a notable clustering of pooled samples from QC, indicating the reliability of the data and the stability of the instrument.

The score plot (Fig. 7A**)** shows that, except for the group isolate of 43, which is inside both positive and negative PC1, most samples are grouped within their respective groups in the same PC. Prominently, PC1 shows clear distinctions in the metabolite composition between groups isolates 12, 42, and 48 in positive PC1 and isolates 6, 35, and 45 in negative PC3. Meanwhile, the group isolates 42 and 43 are also separated from 48 in PC2 showing distinct differences in the content.

The loading plot displays the metabolites that are responsible for the *m/z* values-based separation of each sample group. In Fig. 7B, the loading plot demonstrates the influence of metabolites to the segregation of three distinct clusters (S Table 1 and Fig. 12). It is suggested that samples grouped in the same cluster either contain these specific metabolites or produce them in high abundance. The first cluster is characterized by metabolites P_2763 (*m/z* 547.2164), P_2802 (*m/z* 533.1999), P_2552 (*m/z* 283.0961), P_3136 (*m/z* 561.2314), N_3091 (*m/z* 245.0432), P_2553 (*m/z* 427.1151), P_4855 (*m/z* 299.2397), P_3780 (*m/z* 273.2244), P_3254 (*m/z* 138.0807), P_4627 (*m/z* 273.1961), and P_4740 (*m/z* 274.1914). Meanwhile, cluster 2 is distinguished by higher levels of P_2616 (*m/z* 279.1335), P_2674 (*m/z* 280.1224), P_2712 *(m/z* 236.1755), P_2956 (*m/z* 222.1598), P_2632 (*m/z* 164.0929), P_2679 (*m/z* 213.0691), P_2615 (*m/z* 235.0508), P_2592 (*m/z* 239.0787), P_2977 (*m/z* 284.1966), P_2991 (*m/z* 284.1966), and P_3479 (*m/z* 321.1436). Meanwhile, metabolites P_6944 (*m/z* 391.2028), P_6947 (*m/z* 251.0465), P_6948 (*m/z* 481.2681), P_4674 (*m/z* 357.2031), and P_6954 (*m/z* 413.1847) contribute to the separation of group isolate 10 from the other groups suggested these metabolites were only produced in the actinomycete group isolate 10 compared to others.

Using readily available databases, such as DNP [49], one can dereplicate the secondary metabolites discovered in the crude extracts. This process is commonly employed as a metabolomics plan in the detection of naturally occurring products to help isolate novel bioactive compounds [22, 26] and to explore their various bioactivities. Here, dereplication research was carried out to investigate our actinomycetes’ antimicrobial and anticancer properties. To obtain the most similar matches for our detected metabolites in clusters 1, 2, and 3, we dereplicated the HRMS data through a comparison of their molecular weight and expected molecular formula (MF) with the identified products found in the DNP database and the free online natural product database available at http://www.knapsackfamily.com/knapsack_core/top.php.

The majority of these metabolites in different clusters had either no hits or no microbial hits, reflecting a great possibility of isolating new products from our extracts. Additionally, some of these metabolites were thought to be natural compounds previously recovered from other bacteria or fungi. Interestingly, some molecules from these tentatively identified metabolites demonstrated a varied range of bioactivities, like antitumor and antimicrobial properties, which might clarify the antimicrobial and anticancer potentialities of our selected biological active actinomycetes. For instance, the mass ion peak at *m/z* 138.0807 [M + H]^+^ with expected MF C_16_H_33_N_3_O_9_ had the predicted match of **Sorbistin A2 (1)**, isolated from the bacterium *Pseudomonas sorbicinii* sp. nov. D496-B83 exhibiting moderate but very broad antibacterial activity and inhibiting a majority of aminoglycoside-resistant organisms [50]. Also, the mass ion peak at *m/z* 273.1961 [M + H]^+^ with expected MF C_17_H_24_N_2_O was chemically annotated as **Nb-(5-Methylhexanoyl) tryptamine (2)**, produced by bacterium *Xenorhabdus doucetiae* [51]. Other synthesized tryptamine derivatives produced by *Xenorhabdus doucetiae* displayed potent cytotoxic activity against HL-60 (human acute myeloid leukemia; ACC 3) and L-929 (mouse fibroblast; ACC 2) cell lines. It also exhibited broad insecticidal activity [51].

Additionally, the mass ion peak at *m/z* 283.0961 [M + H]^+^ with expected MF C_17_H_14_O_4_ was dereplicated based on the closest match in databases to be the **Eutypoid B (3)**, previously recovered from marine-derived fungus *Penicillium* sp. KF620 [52] demonstrating good bioactivity against glycogen synthase kinase 3β in the management of type 2 diabetes [53], and as **5**,**7-dimethoxy-4-phenyl coumarin (4)**, extracted from *Streptomyces aureofaciens* CMUAc130 exhibiting cytotoxic activity against Lewis lung carcinoma (LLC) with T/C values of 81.5% (at low dose) and 44.9% (at high dose) and inhibited A427 (human lung cancer cell lines) cell growth by 42% [54]. It also displayed antifungal activity against *Colletotrichum musae* and *Fusarium oxysporum* with a MIC value of 150 µg/ml [55]. This mass ion peak was also supposedly recognized as **3**,**8-dihydroxy-1-propylanthraquinone (5)**, isolated from aquatic-derived bacteria *Streptomyces* sp. FX-58 and B8000 and *Micromonospora rhodorangea* with selected antimicrobial activity, and as **2-Ethyl-1**,**8-dihydroxy-3-methylanthraquinone (6)**, extracted from marine-derived *Streptomyces* sp. FX-58 and B8000 and *Micromonospora rhodorangea* with cytotoxic activity against HL-60 cell line [55–58]. It was also identified as **9-hydroxy micro perfuranone (7)**, isolated from the fungus *Emericella quadrilineata* IFM 42,047 and showed immunomodulatory activity [59].

Furthermore, the mass ion peak at *m/z* 533.1999 [M + H]^+^ with expected MF C_24_H_32_N_6_O_4_S_2_ was annotated as **aerucyclamide B (8)**, separated from bacterium *Microcystis aeruginosa* PCC 7806 displaying anthelminthic activity against *Thamnocephalus platyurus* with LC_50_ value of 33.8 µM [60]. The mass ion peak at *m/z* 279.1335 [M + H]^+^ with expected MF C_14_H_18_N_2_O_4_ was supposedly recognized as **3-Hydroxy-3-[(4-methoxyphenyl)methyl]-1**,**4-dimethyl-2**,**5-piperazine-dione (9)**, previously produced from *Penicillium brevi-compactum* and exhibiting antiviral and cytotoxic activity [61]. Remarkably, the mass ion peak at *m/z* 321.1436 [M + H]^+^ with expected MF C_16_H_20_N_2_O_5_ was dereplicated based on the closest match in databases to be **3’**-**N**-**Formyl Fusarochromanone (10)**, isolated from the fungus *Fusarium equiseti* [62]. Interestingly, its parent compound Fusarochromanone exhibited broad cytotoxic activity [63].

Furthermore, the mass ion peak at *m/z* 251.0465 [M + H]^+^ with expected MF C_13_H_11_ClO_3_ was chemically annotated as **6-Hydroxy pterulone (11)**, previously isolated from the fungus *Mycena galopus*. The parent compound (pterulone) and its derivatives displayed antitumor activity against L1210 (mouse lymphocytic leukemia), HL 60, BHK (baby hamster kidney fibroblasts) and Hela S3 (cervical adenocarcinoma) cell lines with IC_50_ value of more than 100 µg/ml. They also exhibited antifungal activity against *Nadsonia fulvescens*, *Rhodotorula glutinis*, *Saccharomyces cerevisiae* S288c, *Saccharomyces cerevisiae* is1, *Nematospora coryli*, *Paecilomyces variotii*, *Mucor miehei*, *Fusarium oxysporum*, and *Penicillium notatum* [64, 65]. Further, the mass ion peak at *m/z* 391.2028 [M + H]^+^ with expected MF C_23_H_31_ClO_3_ was supposedly recognized as the **5-Chloro ilicicolin B (12)**, which was previously reported from the fungus *Stachybotrys* sp. It is noteworthy that its parent compound Ilicicolin B has antagonistic activity on endothelin receptor, inhibition of tyrosine kinase and immune-suppressive action [53].

**Fig. 8:**
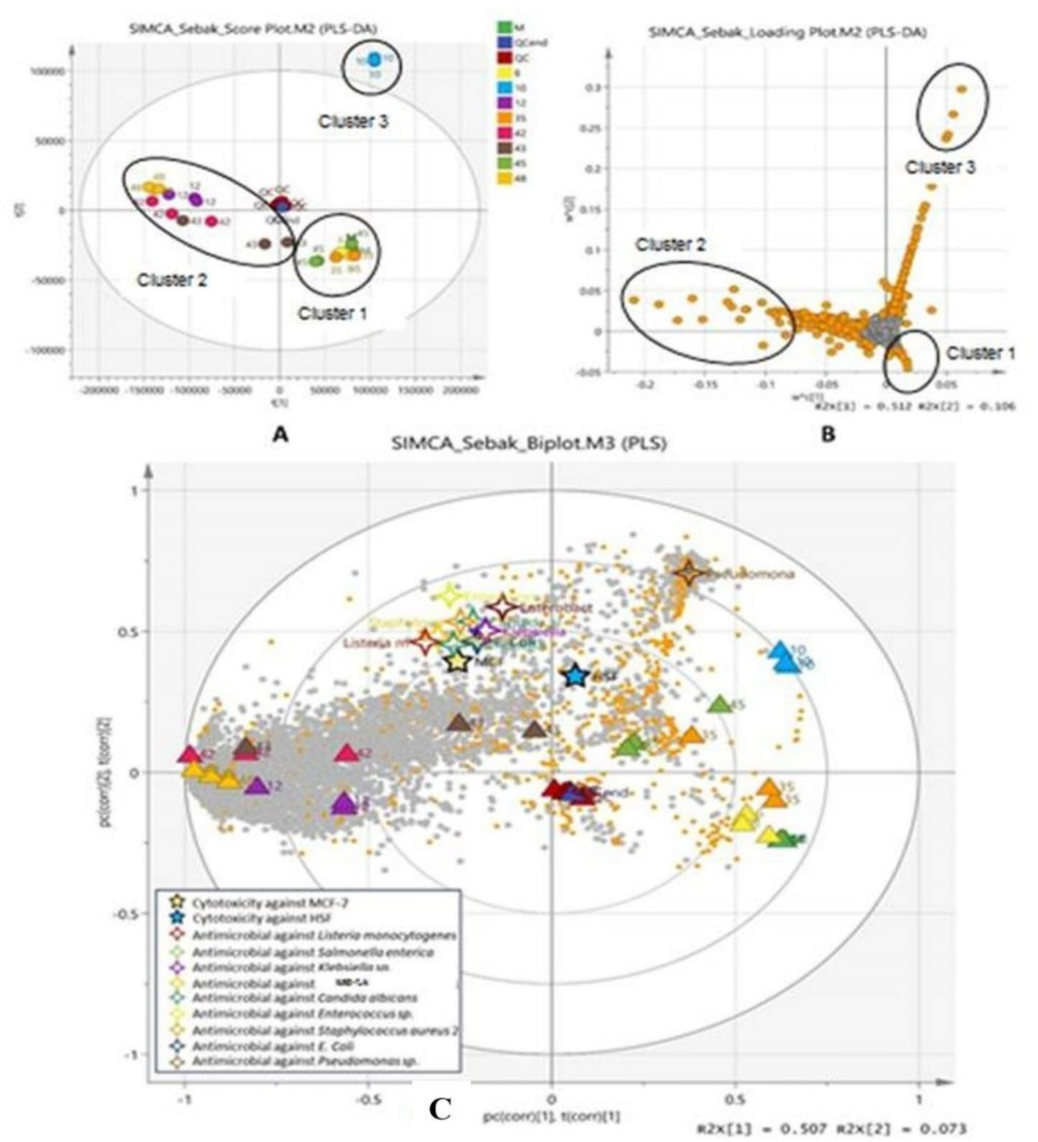
The multivariate variable pattern analysis for the normalized data was conducted using SIMCA software version 14.0. **(A)** Partial Least Squares Discriminant Analysis (PLS-DA) derived from the profiles of different sample groups: Media (M), quality control (QC), and various microbes’ isolation groups: 6, 10, 12, 35, 42, 43, 45, 48. **(B)** PLS-DA loading plot displaying the clustering of metabolite content in different sample groups. (**C**) PLS biplot illustrates the association among biological activities (cytotoxicity and antibacterial activity) as Y-variables and metabolites as X-variables. Orange-colored clusters in the loading plot and biplot are metabolites with VIP > 1

Partial least squares-discriminant analysis (PLS-DA) is used to reduce external variables and further improve our understanding of the identified metabolite patterns in various microbes’ samples, as seen in Fig. 8**(A-C)**. This analysis focuses on increasing the distinction between groups of observations to improve classification and expection by utilizing the discovered metabolites. All samples in the score plot are distributed inside the 95% Hotelling T2 ellipse, with data variation for PC1 and PC2 at 51.2% and 10.6%, respectively, except for the microbial sample isolation 10 which becomes an outlier. The PLS-DA model generated 13 principal components (Comp [1]–Comp [13]). The values of R2X, R2Y, and Q2 were determined to be 0.924, 0.874, and 0.484, respectively. To achieve a model that fits well, it is desirable for the R2 number, which measures how well the model fits the data, to be near to 1. Additionally, the Q2 value, which indicates how well the model predicts new data, should be greater than 0.5 to demonstrate high predictability. A low Q2 score suggests that the data contains a significant amount of noise or that the model is heavily influenced by a small number of distributed outliers.

The score plot in Fig. 8A indicates that the cluster 1 sample is notably different from the cluster 2 sample group based on their position in negative and positive PC1, respectively. The cluster 1 sample group was distinguished from the cluster 2 group in PC2 based on their positive and negative PC2 scores, respectively. All replicate samples within every group did not exhibit a distinct difference except for isolate group 43, consistent with the results of the PCA model. The loading plot in Fig. 8B shows the distribution of metabolites respective to the samples. The orange-colored metabolites are metabolites with a variance of importance (VIP) greater than 1.

The PLS biplot in Fig. 8C showed an overlay of both the score plot and loading plot visualizing the relationship between metabolites, sample groups and biological activity. The antimicrobial and cytotoxicity against MCF-7 cell lines were shown strongly correlated with cluster 2 of the sample groups (sample group isolate numbers 12, 42, 43 and 48). Dereplication analysis identified two known compounds in this cluster. One was tentatively identified as 3-Hydroxy-3-[(4-methoxyphenyl)methyl]-1,4-dimethyl-2,5-piperazine-dione, a compound previously reported to possess antiviral and cytotoxic properties [61]. The other was putatively identified as 3’-N-Formyl Fusarochromanone [62]. Although no studies have reported its antimicrobial or cytotoxic effects, its parent compound, Fusarochromanone, is known to exhibit broad cytotoxic activity [63]. It may be postulated that the observed antimicrobial and cytotoxic effects are attributable to the presence of these compounds. Cluster 1, comprising isolates 6, 35, and 45, exhibited a strong correlation with cytotoxicity against normal human skin fibroblast (HSF) cell lines. This suggests that the extracts, when tested at their IC₅₀ concentrations, were cytotoxic to normal cells, indicating the presence of highly potent cytotoxic compounds. Among the annotated compounds, one was identified as Nb-(5-Methylhexanoyl) tryptamine, whose derivatives have been reported to exhibit strong cytotoxic activity not only against HL-60 (human acute myeloid leukemia; ACC 3) but also against L-929 (normal mouse fibroblast; ACC 2) cell lines. Another annotated compound, 5,7-dimethoxy-4-phenyl coumarin, has been reported to display cytotoxic effects against Lewis lung carcinoma (LLC), with T/C values of 81.5% at low dose and 44.9% at high dose, and to inhibit the growth of A427 (human lung cancer) cell lines by 42% [51].

**Fig. 9:**
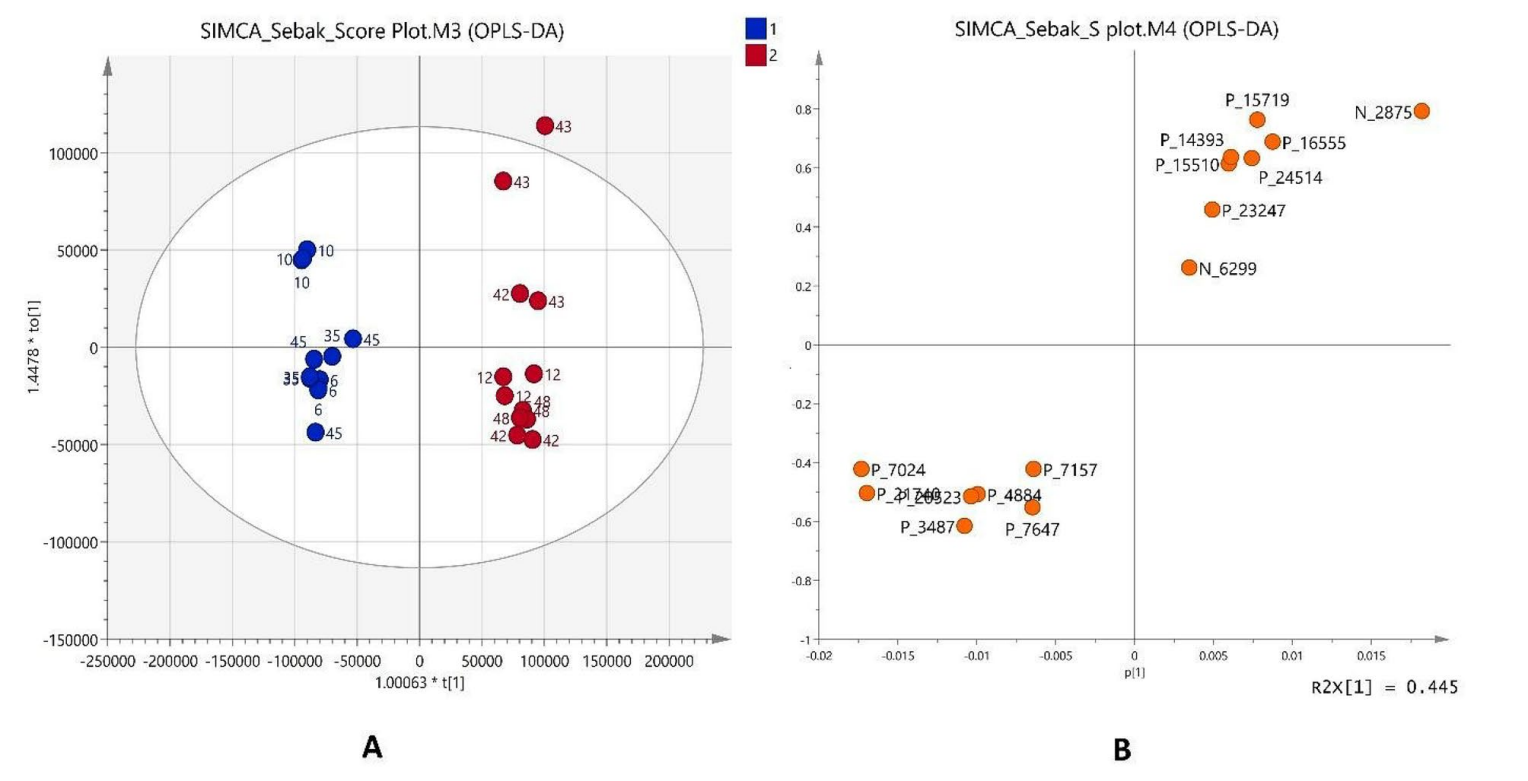
The multivariate variable pattern analysis for the normalized data was conducted using SIMCA software version 14.0. **(A)** Orthogonal Partial Least Squares Discriminant Analysis (OPLS-DA) derived from the LC-MS profiles of different sample groups: Media (M), quality control (QC), and different microbes’ isolation groups: 6, 10, 12, 35, 42, 43, 45, 48. **(B)** S-plot displaying the metabolites according to the importance of variables which are accountable for driving the separation of samples

The OPLS-DA, a supervised model depicted in Fig. 9, enables the filtration of orthogonal metabolite variables unrelated to categorical variables. This method was applied to discern differences in metabolic profiles among groups and highlights the variations in metabolites among these groups, thereby augmenting classification outcomes. The loading plot’s PC1 illustrates a variance of 44.5% and 11.1% for PC1 and PC2, respectively. High R2X (cum), R2Y(cum), and Q2 (cum) values of 0.628, 0.983, and 0.915, respectively, affirm the robust fit and predictability of the OPLS-DA model.

**Fig. 10:**
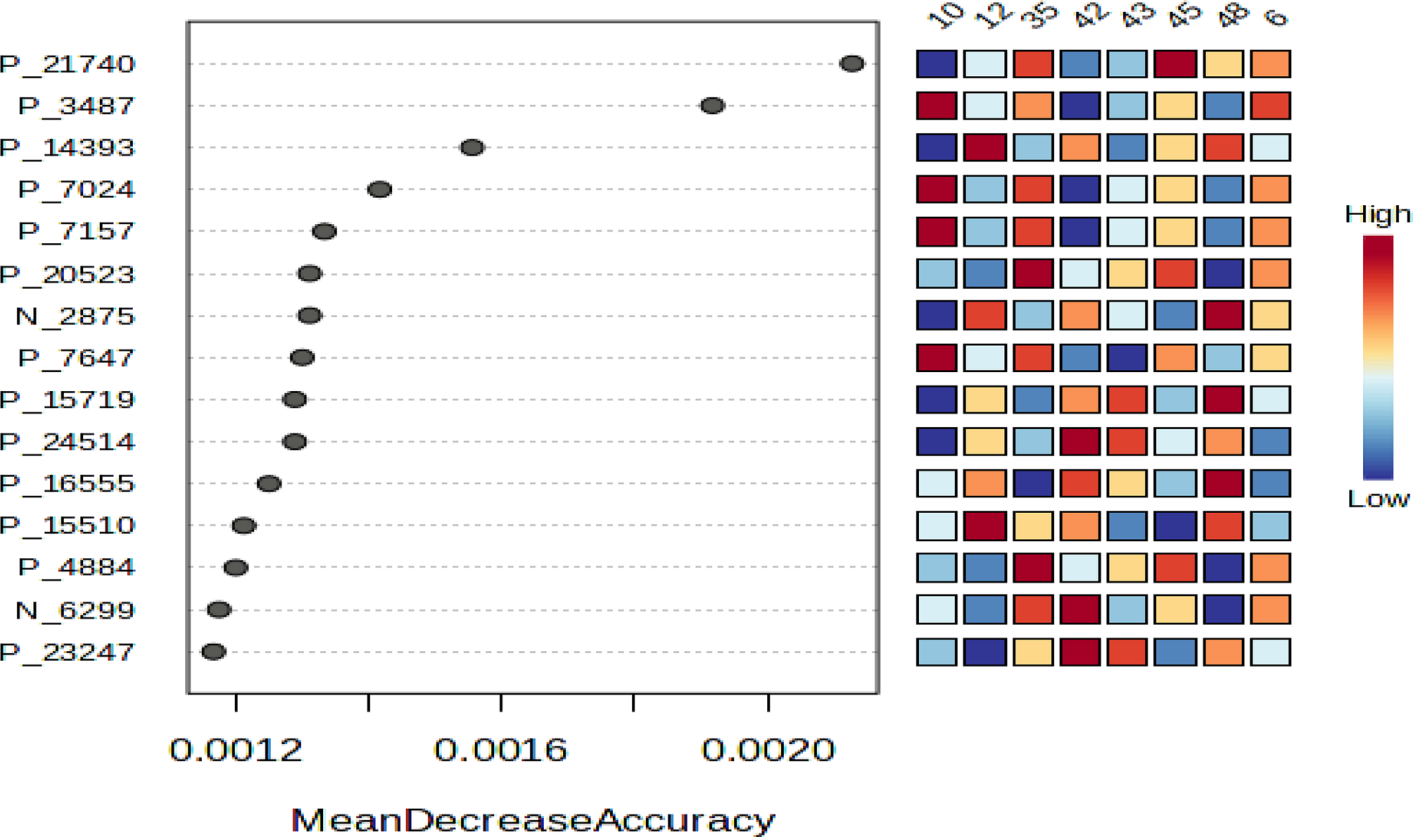
Random Forest analysis was done using Metaboanalyst 6.0. The top 15 metabolites with the greatest discriminating power between two distinct groups are listed. Red fields indicate a high concentration, whereas blue fields indicate a low concentration of the same metabolite

The OPLS-DA score plot in Fig. 9A delineates two distinct clusters exhibiting separation from each other by PC1, indicating dissimilarities in metabolites between these groups. The Random Forest Analysis was done to determine the 15 most significant metabolites in discriminating between different groups as shown in Fig. 10. Metabolites P_21740 (*m/z* 271.2088), P_3487 (*m/z* 739.4531), P_14393 (*m/z* 423.2273), P_7024 (*m/z* 454.2112), P_7157 (*m/z* 541.2896), P_20523 (*m/z* 703.2043), N_2875 (*m/z* 132.0564), P_7647 (*m/z* 185.1075), P_15719 (*m/z* 371.1963), P_24514 (*m/z* 315.1330), P_16555 (*m/z* 222.0074), P_15510 (*m/z* 368.1696), P_4884 (*m/z* 584.2706), N_6299 (*m/z* 489.1049) and P_23247 (*m/z* 1009.6293) are the metabolites significantly contribute to the difference in sample groups. The ensuing results were then visually depicted in a loadings S-plot in Fig. 9B, displaying the covariance (p) against the correlation (pcorr). S-plot was utilized to analyze the significance of variables (features) for differentiating between the various groups.

**Fig. 11:**
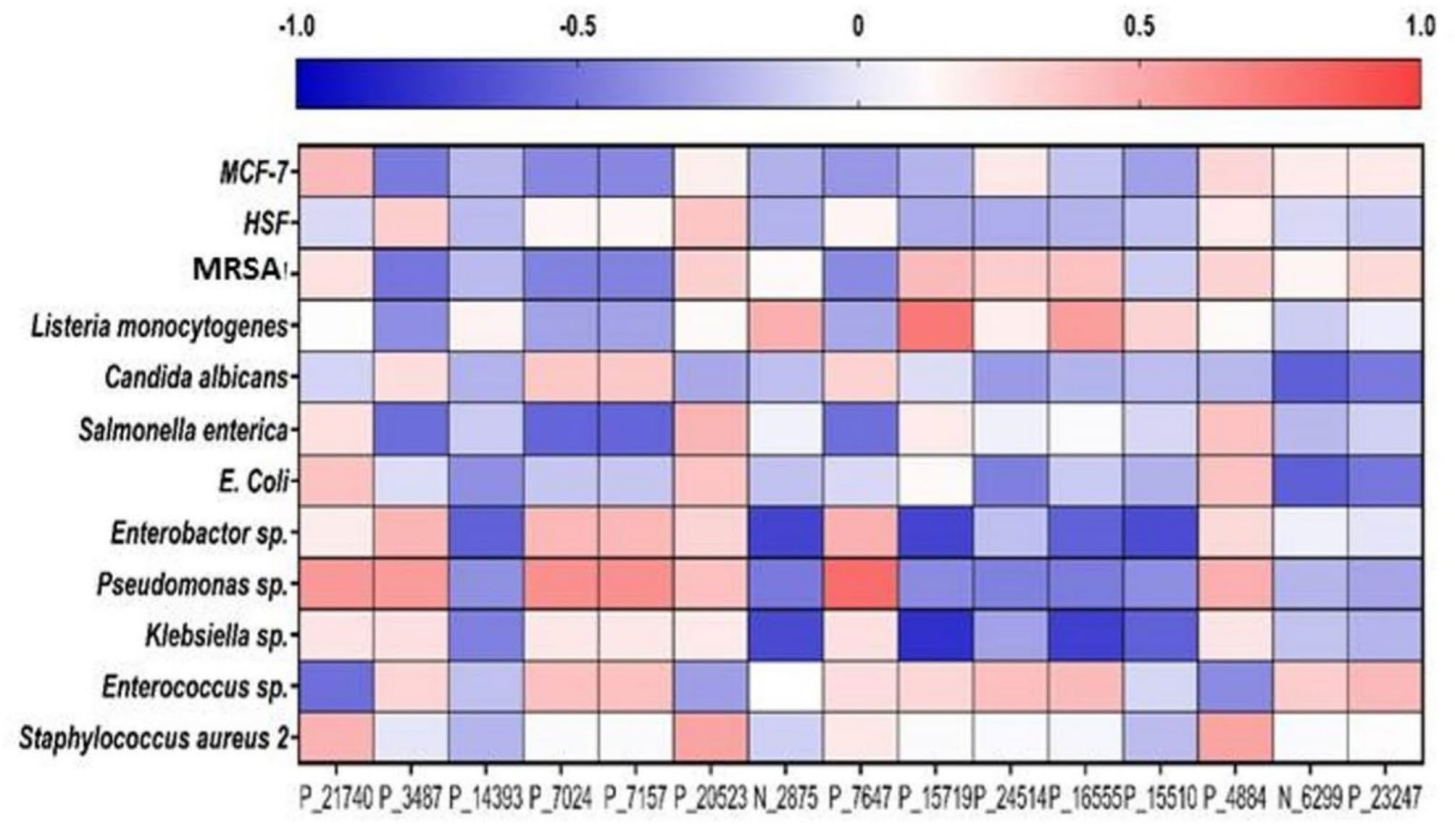
Pearson correlation of significantly different metabolites (*p* < 0.05) was executed using Graph Pad Prism 9.0 with cytotoxicity activity against MCF-7 and HSF cell lines and microbial activity against different microbe groups. The values of Pearson’s correlation coefficient (r) are indicated by each square with red representing positive (0 < *r* < 1) and blue representing negative (− 1 < *r* < 0) correlations

Furthermore, a Pearson Correlation Analysis was executed to decide the statistical association among the patterns of the corresponding biological activity including cytotoxicity and antimicrobial activity and the 15 metabolites that were discovered to be significantly different. Figure 11 shows that most of the metabolites show a negative correlation to cytotoxicity against both MCF and HSF cell lines. Metabolites P_21740 (*m/z* 271.2088), P_20523 (*m/z* 703.2043), P_24514 (*m/z* 315.1330), P_4884 (*m/z* 584.2706), N_6299 (*m/z* 489.1049), and P_23247 (*m/z* 1009.6293) are positively correlated to cytotoxicity against MCF-7 cell lines and metabolites P_3487 (*m/z* 739.4531), P_7024 (*m/z* 454.2112), P_7157 (*m/z* 541.2896), P_20523 (*m/z* 703.2043), P_7647 (*m/z* 185.1075) and P_4884 (*m/z* 584.2706) are positively correlated to cytotoxicity against HSF cell lines. Meanwhile, the correlation ranges widely from negative correlation to positive correlation to microbial activity against different microbes. Most metabolites show a positive correlation to microbial effects against the drug-resistant *Enterobacter* sp., *Pseudomonas* sp., *Klebsiella* sp. and *Enterococcus* sp. Meanwhile, most metabolites show a negative correlation to the MRSA, *Listeria monocytogenes*, *Candida albicans*, *Salmonella enterica*, *E. coli* and drug-resistant *Staphylococcus aureus* 2.

**Fig. 12:**
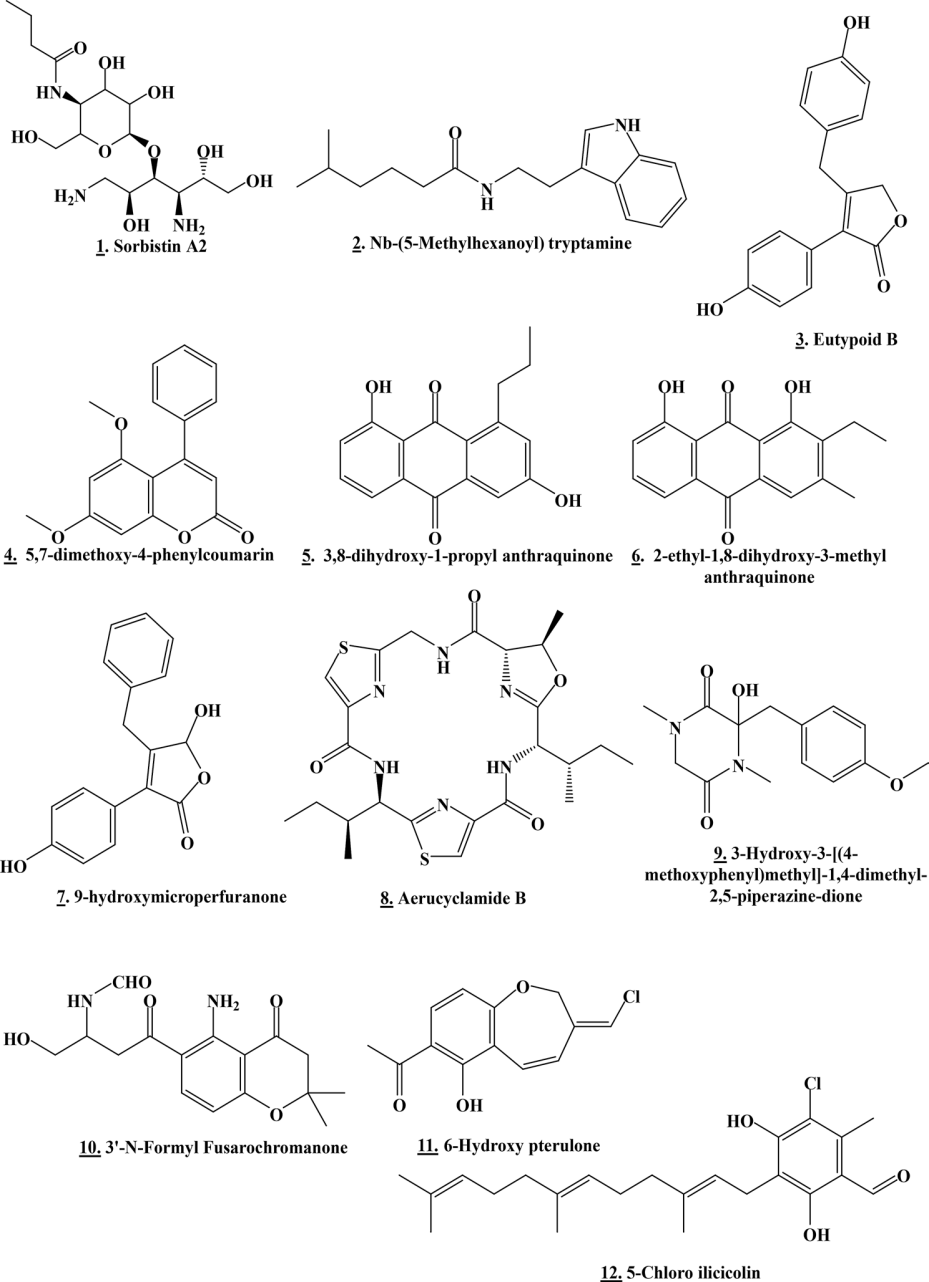

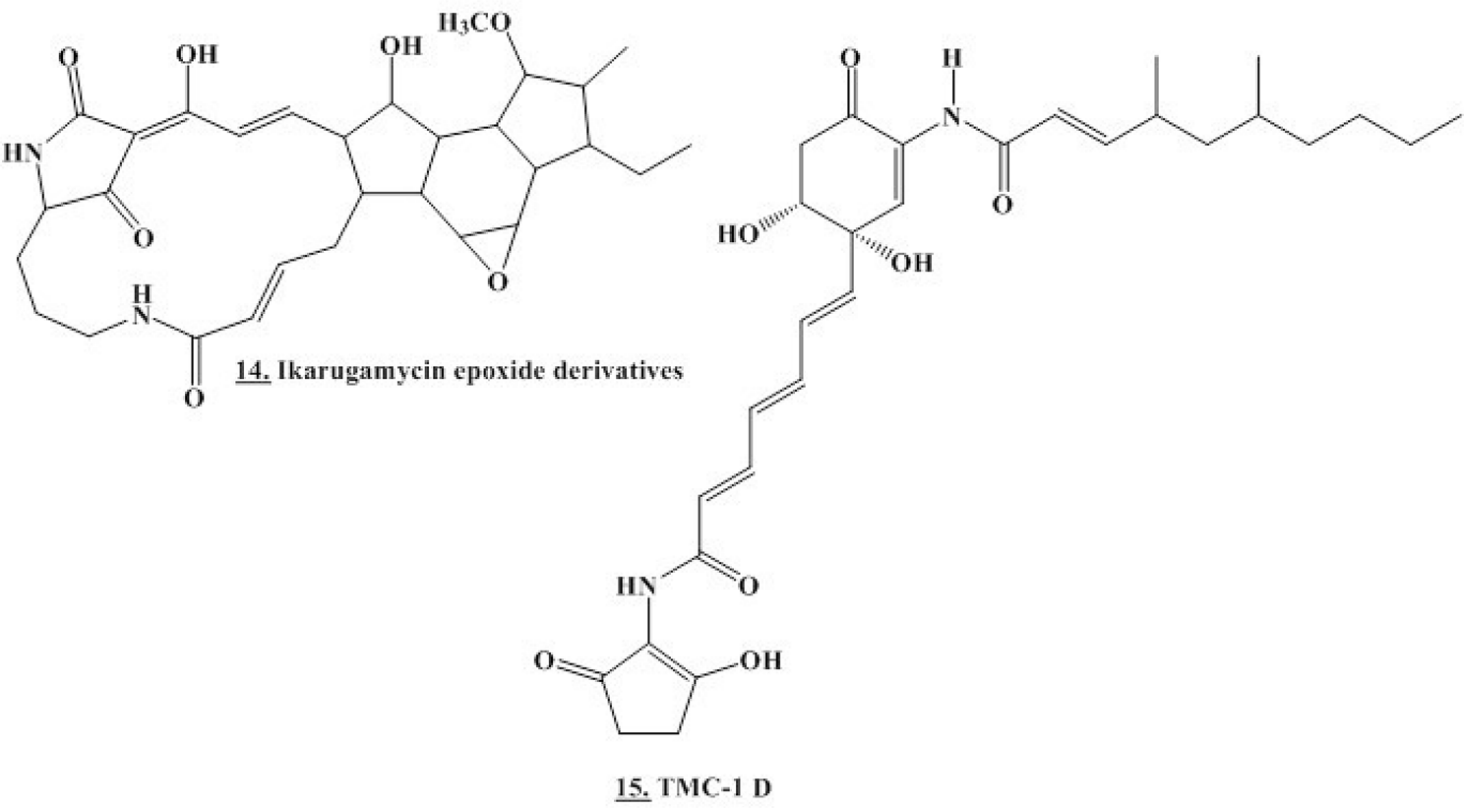
Chemical structure of the closest matches to the metabolites identified from the actinomycetes by molecular weight from databases of natural products (S1 and S2 Table). The structure of the Antibiotic C 13,648 (13) is unavailable

A dereplication study of these fifteen interesting metabolites using the same strategy mentioned above demonstrated that only two metabolites had microbial hits. Other features were annotated only to the level of molecular formula and remained unknown (S Table 2 and Fig. 12).The mass ion peak at *m/z* 370.1890 [M + H]^+^ with expected MF C_21_H_26_N_2_O_4_ was chemically annotated as **Antibiotic C 13,648 (13)**, separated from the actinobacterium *Streptomyces antibioticus* C13648 and exhibited antibacterial activity with inhibit plaque formation. Outstandingly, the mass ion peak at *m/z* 541.2896 [M + H]^+^ with expected MF C_30_H_40_N_2_O_7_ was supposedly recognized as **Ikarugamycin 26R-Methoxy**,** 16ξ-hydroxy**,** 4β**,**5β-epoxide (14)**, isolated from the actinomycete *Streptomyces* sp. Other Ikarugamycin derivatives produced by *Streptomyces* sp. demonstrated antimicrobial activity against *Arthrobacter aurescens* DSM 20,166, *Arthrobacter globiformis* DSM 20,124, *Arthrobacter pascens* DSM 20,545, *Arthrobacter oxydans* DSM 6612, *Staphylococcus aureus* DSM 20,231, *Bacillus subtilis* DSM 10, *Streptomyces viridochromogenes* Tu 57, *Botrytis cinerea* Tu 157, *Mucor hiemalis* Tu 179/Tu 180 and *Paecilomyces variotti* Tu l 37. They also displayed cytotoxic activity against HMO2 (gastric carcinoma), MCF-7, HepG2 and Huh 7 (human hepatoma-derived) cell lines [66]. This mass ion peak was also noted as **TMC-1 D (15)**, previously isolated from *Streptomyces* sp. A-230 showing antitumor properties against HCT-116, OVCAR-3 (human ovarian adenocarcinoma), SW480 (human colon adenocarcinoma), WiDr (human colon adenocarcinoma), Saos-2 (human osteogenic sarcoma), HL-60, HeLa S3 and P388Dl (murine lymphoid neoplasm) cells lines with an IC_50_ value of 6.8, 11, 5.9. 9.3, 6, 12, 8.3 and 3.2 µg/mL respectively [67].

### NMR fingerprinting and data analysis

**Fig. 13:**
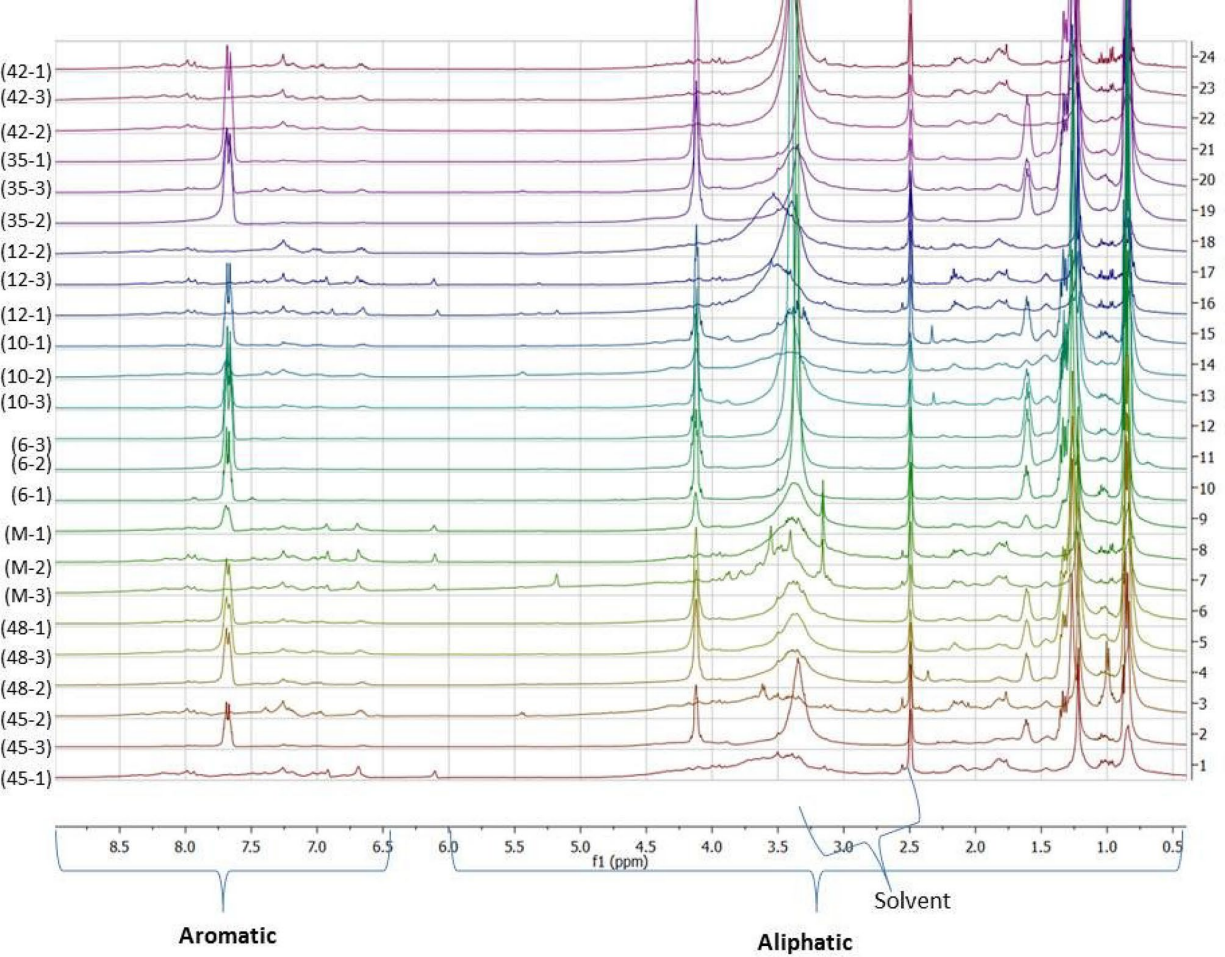
Stacked ^1^H NMR spectra of selected bioactive marine actinomycetes showing peaks in both aliphatic and aromatic regions

**Fig. 14:**
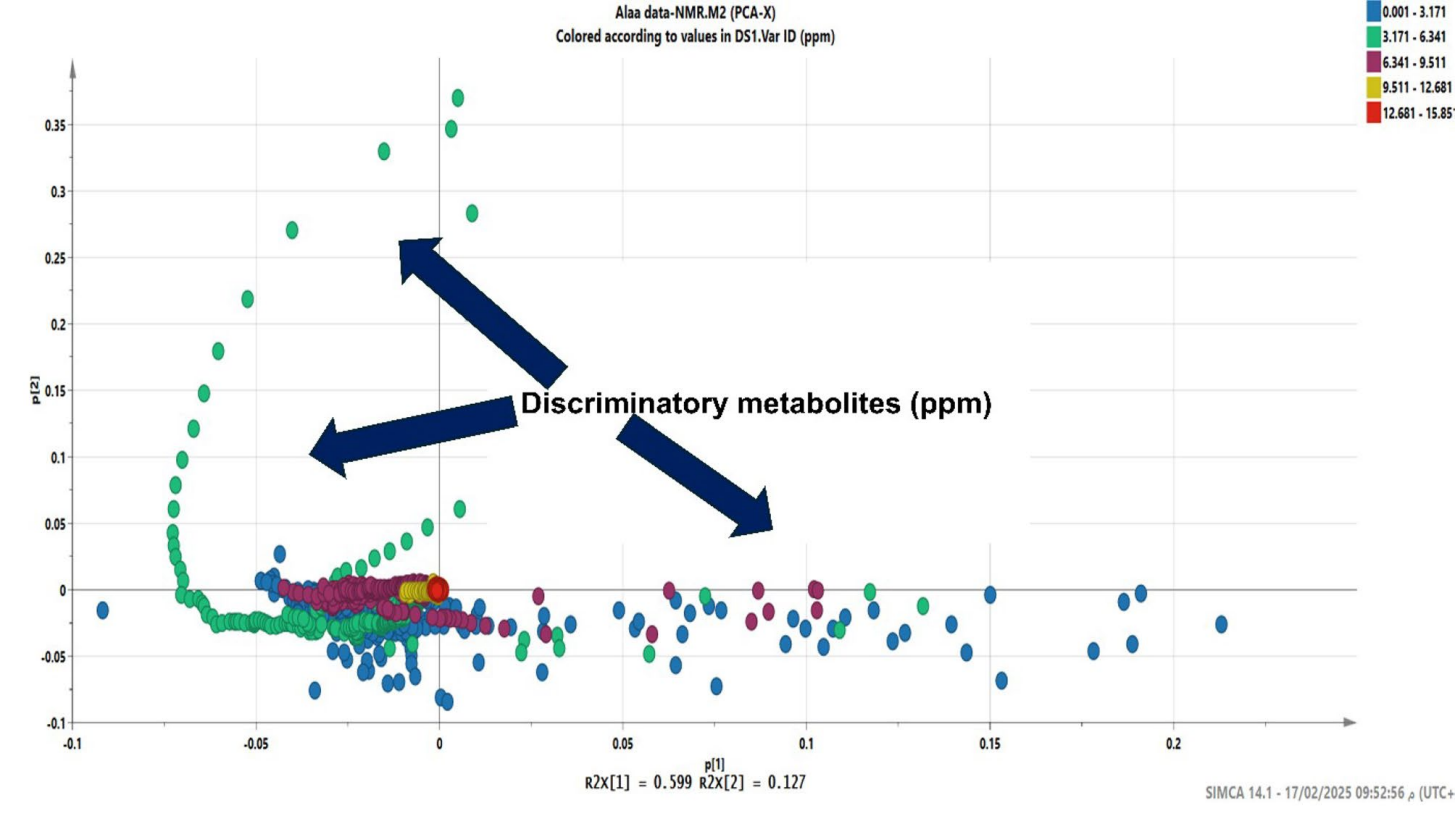
PCA loading plot of the ^1^H NMR chemical shifts data in ppm of selected bioactive marine actinomycetes with highlighting of the interesting discriminatory metabolites

As presented in Fig. 13, the ^1^H NMR fingerprinting of our selected bioactive actinomycetes showed promising peaks in both aliphatic (0–6 ppm) and aromatic (6.5-9 ppm) regions indicating the richness of their chemical profiles similar to several previous reports on marine actinomycetes [68, 69]. Intriguingly, this was also confirmed by the MVDA of ^1^H NMR data. The PCA model had R^2^ value of 0.868 and Q^2^ value of 0.737 from 5 components which indicates the goodness of the model. The loadings plot of the PCA of ^1^H NMR data revealed the widespread distribution of the NMR peaks with various chemical shifts, whereas the discriminating metabolites which led to separation of extracts’ NMR profiles were distributed among the aliphatic (0–6 ppm) and aromatic peaks (6.5-9 ppm) (Fig. 14). This result highlights the chemical complexity of the actinobacterial extracts. In addition, the NMR MVDA confirmed the variability of the interesting metabolites produced by our extracts which matches the results of the LC-MS MVDA and dereplication analysis.

## Conclusions

To chemically profile actinomycete isolates for isolate prioritizing and chemical separation, the current work emphasizes the significance of integrating dereplication and metabolomics data analysis, containing the application of unsupervised and supervised MVDA. Understanding how secondary metabolites underpin various bioactivities may also be aided by the use of a multivariate analysis and dereplication-based strategy. MVDA of the high-resolution mass spectrometry (HRMS) data identified isolate 10 as an outlier, rich in diverse secondary metabolites. Dereplication revealed numerous metabolites as 3-Hydroxy-3-[(4-methoxyphenyl)methyl]-1,4-dimethyl-2,5-piperazine-dione, 3’-N-Formyl Fusarochromanone and Nb-(5-Methylhexanoyl) tryptamine with potential antimicrobial and anticancer activity, confirmed by NMR data highlighting their complex chemical profiles. It may be postulated that the observed antimicrobial and cytotoxic effects are attributable to the presence of these compounds. Additionally, a metabolomics-guided strategy backed by dereplication and other bioassay results may make it more likely to target and, consequently, isolate novel bioactive natural compounds from various sources, including *Streptomyces* and *Brevibacterium*. Furthermore, our work found that Egyptian marine actinomycetes are still a good source for separating new bioactive natural chemicals, especially when metabolomics is used rather than conventional bioassay-guided methods.

## Electronic supplementary material

Below is the link to the electronic supplementary material.

Supplementary Material 1

## Data Availability

No datasets were generated or analysed during the current study.
