# Supplementary material for: Screening of Red Sea- and Mediterranean Sea-derived Actinomycetes for Antimicrobial and Antitumor activities: LC-ESI-HRMS-based Metabolomics Study
Source: Microb Cell Fact. 2025 Jun 18;24:136. doi: 10.1186/s12934-025-02759-0 (PMC12175458; doi:10.1186/s12934-025-02759-0)
Supplement: Supplementary file 1 — Supplementary Material 1 [file 12934_2025_2759_MOESM1_ESM.docx]

**Screening of Red Sea- and Mediterranean Sea-derived Actinomycetes for Antimicrobial and Antitumor activities: LC-ESI-HRMS-based Metabolomics Study**

Mahmoud A. Abdel-Razik^1*^, Ahmed F. Azmy^1^, Tarek Dishisha^1^, Ahmed O. El-Gendy^1^, Adlin Afzan^2^, Nurkhalida Kamal^3^, Ahmed Tawfike^4^, and Mohamed Sebak^1^

^1^Department of Pharmaceutical Microbiology and Immunology, Faculty of Pharmacy, Beni-Suef University, Beni-Suef 62514, Egypt.

^2^Phytochemistry Unit, Herbal Medicine Research Centre, Institute for Medical Research, National Institutes of Health, Ministry of Health Malaysia, 40170 Shah Alam, Selangor, Malaysia

^3^Institute of Systems Biology, Universiti Kebangsaan Malaysia, 43600, UKM, Bangi, Selangor, Malaysia

^4^Department of Pharmacognosy, Faculty of Pharmacy, Helwan University, 11795 Cairo, Egypt

^*^Correspondence:

* dr_mahmoudalaa_91@yahoo.com

Tel.: +201143347137

**S Table 1:** Dereplication analysis of the secondary metabolites of the three clusters of metabolites extracted from the PCA in the MVDA (Figure 7). *m/z* = mass/charge ratio, RT = retention time, MW = molecular weight). Ionization mode is designated on MZmine ID as P for positive mode [M+H]^+^ and N for negative mode [M-H]^-^.

| **Hits in natural products databases / Microbial source** | **Predicted formula** | **MW** | ***m/z*** | **RT**  **(min)** | **MZmine**  **ID** | **Cluster** |
| --- | --- | --- | --- | --- | --- | --- |
| No microbial hits | C_6_H_15_O_8_P | 246.0504 | 245.0432 | 0.93 | N_3091 | **1** |
| No microbial hits | C_13_H_25_N_13_O_3_  C_28_H_29_NO_2_  C_12_H_29_N_9_O_7_ | 137.0735 | 138.0807 | 10.29 | P_3254 |  |
| Sorbistin A2/ *Pseudomonas sorbicinii* sp. nov. D496-B83 | C_16_H_33_N_3_O_9_ |  |  |  |  |  |
| Nb-(5-Methylhexanoyl) tryptamine/ *Xenorhabdus* *doucetiae* | C_17_H_24_N_2_O | 272.1888 | 273.1961 | 10.40 | P_4627 |  |
| No microbial hits | C_16_H_23_N_3_O | 273.1841 | 274.1914 | 11.53 | P_4740 |  |
| No microbial hits | C_20_H_23_ClO_8_ | 426.1078 | 427.1151 | 11.88 | P_2553 |  |
| Eutypoid B/marine-derived *Penicillium* sp. KF620 | C_17_H_14_O_4_ | 282.0889 | 283.0961 | 11.88 | P_2552 |  |
| 5,7-dimethoxy-4-phenylcoumarin/ *Streptomyces* *aureofaciens* CMUAc130 |  |  |  |  |  |  |
| 3,8-Dihydroxy-1-propylanthraquinone/ marine-derived *Streptomyces* sp. FX-58 and B8000 and *Micromonospora* *rhodorangea* |  |  |  |  |  |  |
| 2-Ethyl-1,8-dihydroxy-3-methylanthraquinone/ marine-derived *Streptomyces* sp. FX-58 and B8000 and *Micromonospora* *rhodorangea* |  |  |  |  |  |  |
| 9-hydroxymicro  perfuranone/ *Emericella* *quadrilineata* IFM 42047 |  |  |  |  |  |  |
| No microbial hits | C_22_H_72_N_32_O_2_  C_39_H_88_N_6_O_11_  C_37_H_76_N_20_O  C_52_H_80_N_8_  C_40_H_84_N_10_O_7_  C_36_H_80_N_16_O_5_  C_56_H_84_N_2_O_2_  C_51_H_84_N_4_O_4_  C_41_H_80_N_14_O_3_  C_18_H_68_N_38_ | 272.2171 | 273.2244 | 12.23 | P_3780 |  |
| Aerucyclamide B/ *Microcystis* *aeruginosa* PCC 7806 | C_24_H_32_N_6_O_4_S_2_ | 532.1926 | 533.1999 | 12.35 | P_2802 |  |
| No microbial hits | C_27_H_82_N_28_O_6_  C_58_H_86_N_8_  C_42_H_86_N_16_O_5_  C_43_H_82_N_20_O  C_45_H_94_N_6_O_11_  C_57_H_90_N_4_O_4_  C_24_H_74_N_38_  C_28_H_78_N_32_O_2_  C_41_H_90_N_12_O_9_  C_13_H_74_N_44_O_3_ | 298.2324 | 299.2397 | 12.50 | P_4855 |  |
| No microbial hits | C_25_H_34_N_6_O_4_S_2_  C_28_H_34_O_11_ | 546.2091 | 547.2164 | 12.50 | P_2763 |  |
| No microbial hits | C_29_H_36_O_11_ | 560.2241 | 561.2314 | 12.66 | P_3136 |  |
| No microbial hits | C_7_H_9_N_5_ | 163.0856 | 164.0929 | 0.94 | P_2632 | **2** |
| 3-Hydroxy-3-[(4-methoxyphenyl)methyl]-1,4-dimethyl-2,5-piperazine-dione/ *Penicillium brevi-compactum* | C_14_H_18_N_2_O_4_ | 278.1262 | 279.1335 | 2.81 | P_2616 |  |
| No microbial hits | C_24_H_63_N_31_O_4_  C_41_H_79_N_5_O_13_  C_39_H_67_N_19_O_3_  C_42_H_75_N_9_O_9_  C_54_H_71_N_7_O_2_  C_27_H_71_N_21_O_10_  C_23_H_67_N_27_O_8_  C_58_H_75_NO_4_  C_40_H_83_NO_17_  C_38_H_71_N_15_O_7_ | 283.1893 | 284.1966 | 4.69 | P_2991 |  |
| No microbial hits | C_12_H_19_N_3_O | 221.1525 | 222.1598 | 4.83 | P_2956 |  |
| No microbial hits | C_24_H_63_N_31_O_4_  C_41_H_79_N_5_O_13_  C_39_H_67_N_19_O_3_  C_42_H_75_N_9_O_9_  C_54_H_71_N_7_O_2_  C_23_H_67_N_27_O_8_  C_25_H_59_N_35_  C_27_H_71_N_21_O_10_  C_58_H_75_NO_4_  C_40_H_83_NO_17_ | 283.1893 | 284.1966 | 4.91 | P_2977 |  |
| 3'-N-Formyl Fusarochromanone/ *Fusarium equiseti* | C_16_H_20_N_2_O_5_ | 320.1364 | 321.1436 | 6.28 | P_3479 |  |
| No microbial hits | C_23_H_63_N_17_O_8_  C_38_H_67_N_5_O_7_  C_39_H_63_N_9_O_3_  C_24_H_59_N_21_O_4_  C_20_H_55_N_27_O_2_  C_37_H_71_NO_11_  C_35_H_59_N_15_O  C_50_H_63_N_3_  C_25_H_55_N_25_  C_27_H_67_N_11_O_10_ | 235.1682 | 236.1755 | 6.31 | P_2712 |  |
| No microbial hits | NA | 238.0714 | 239.0787 | 6.38 | P_2592 |  |
| No microbial hits | C_9_H_12_N_2_O_2_S | 212.0618 | 213.0691 | 6.89 | P_2679 |  |
| No microbial hits | C_28_H_10_N_22_O_3_  C_30_H_22_N_8_O_13_  C_13_H_6_N_34_O_4_  C_15_H_18_N_20_O_14_  C_17_H_30_N_6_O_24_  C_16_H_34_N_2_O_28_  C_14_H_22_N_16_O_18_  C_12_H_10_N_30_O_8_  C_46_H_22_O_8_  H_14_N_32_O_15_ | 234.0435 | 235.0508 | 6.89 | P_2615 |  |
| No microbial hits | C_29_H_55_N_7_O_21_  C_27_H_43_N_21_O_11_  C_25_H_31_N_35_O  C_58_H_47_NO_5_  C_12_H_39_N_33_O_12_  C_14_H_51_N_19_O_22_  C_42_H_47_N_9_O_10_  C_40_H_35_N_23_  C_43_H_43_N_13_O_6_  C_11_H_43_N_29_O_16_ | 279.1151 | 280.1224 | 9.19 | P_2674 |  |
| 6-Hydroxy pterulone/ *Mycena galopus* | C_13_H_11_ClO_3_ | 250.0393 | 251.0465 | 12.66 | P_6947 | **3** |
| No microbial hits | NA | 412.1774 | 413.1847 | 12.67 | P_6954 |  |
| 5-Chloro ilicicolin/ *Stachybotrys* sp. | C_23_H_31_ClO_3_ | 390.1955 | 391.2028 | 12.68 | P_6944 |  |
| No microbial hits | C_18_H_32_N_2_OS_2_ | 356.1958 | 357.2031 | 12.72 | P_4674 |  |
| No microbial hits | C_65_H_120_N_2_O_32_  C_63_H_108_N_16_O_22_  C_61_H_96_N_30_O_12_  C_59_H_84_N_44_O_2_  C_32_H_104_N_36_O_28_  C_46_H_92_N_42_O_13_  C_30_H_92_N_50_O_18_  C_48_H_104_N_28_O_23_  C_50_H_116_N_14_O_33_  C_78_H_112_N_4_O_21_ | 480.2608 | 481.2681 | 12.73 | P_6948 |  |

**S Table 2:** Dereplication analysis of the 15 most significant metabolites in discriminating between different groups in the Random Forest Analysis (Figure 10). *m/z* = mass/charge ratio, RT = retention time, MW = molecular weight). Ionization mode is designated on MZmine ID as P for positive mode [M+H]^+^ and N for negative mode [M-H]^-^.

| **Hits in natural products databases / Microbial source** | **Predicted formula** | **MW** | ***m/z*** | **RT**  **(min)** | **MZmine**  **ID** |
| --- | --- | --- | --- | --- | --- |
| No microbial hits | C_5_H_21_N_17_O_5_  C_20_H_25_N_5_O_4_  C_21_H_21_N_9_  C_6_H_17_N_21_O  C_19_H_29_NO_8_ | 133.0637 | 132.0564 | 0.86 | **N_2875** |
| No microbial hits | C_18_H_25_NO_7_ | 367.1623 | 368.1696 | 6.27 | **P_15510** |
| No microbial hits | C_23_H_22_O_12_ | 490.1122 | 489.1049 | 6.61 | **N_6299** |
| Antibiotic C 13648/ *Streptomyces antibioticus* C13648 | C_21_H_26_N_2_O_4_ | 370.1890 | 371.1963 | 6.84 | **P_15719** |
| No microbial hits | C_17_H_18_N_2_O_4_ | 314.1257 | 315.1330 | 7.33 | **P_24514** |
| No microbial hits | C_13_H_17_N_3_O_28_  C_11_H_5_N_17_O_18_  C_27_H_5_N_9_O_13_  C_26_H_9_N_5_O_17_  C_14_H_13_N_7_O_24_  C_10_H_9_N_13_O_22_  C_25_H_13_NO_21_  C_15_H_9_N_11_O_20_  C_38_H_5_N_3_O_10_  H_5_N_23_O_21_ | 221.0001 | 222.0074 | 7.94 | **P_16555** |
| No microbial hits | C_12_H_12_N_2_ | 184.1003 | 185.1075 | 11.13 | **P_7647** |
| No microbial hits | C_157_H_353_N_47_O_8_  C_129_H_248_N_42_O_40_  C_131_H_260_N_28_O_50_  C_188_H_357_N_27_O_2_  C_190_H_369_N_13_O_12_  C_146_H_264_N_16_O_49_  C_144_H_252_N_30_O_39_  C_162_H_264_N_8_O_44_  C_174_H_369_N_21_O_17_  C_172_H_357_N_35_O | 1008.6221 | 1009.6293 | 11.66 | **P_23247** |
| No microbial hits | NA | 422.2200 | 423.2273 | 11.68 | **P_14393** |
| No microbial hits | C_22_H_66_N_32_O_2_  C_39_H_82_N_6_O_11_  C_37_H_70_N_20_O  C_40_H_78_N_10_O_7_  C_52_H_74_N_8_  C_25_H_74_N_22_O_8_  C_21_H_70_N_28_O_6_  C_56_H_78_N_2_O_2_  C_36_H_74_N_16_O_5_  C_41_H_74_N_14_O_3_ | 270.2016 | 271.2088 | 12.01 | **P_21740** |
| No microbial hits | C_100_H_191_N_43_O_7_  C_102_H_203_N_29_O_17_  C_104_H_215_N_15_O_27_  C_74_H_98_N_24_O_49_  C_133_H_207_N_9_O_11_  C_119_H_219_N_3_O_26_  C_117_H_207_N_17_O_16_  C_115_H_195_N_31_O_6_  C_56_H_86_N_46_O_44_  C_87_H_199_N_41_O_18_ | 702.1970 | 703.2043 | 12.11 | **P_20523** |
| Ikarugamycin; 26R-Methoxy, 16ξ-hydroxy, 4β,5β-epoxide/ *Streptomyces* sp. | C_30_H_40_N_2_O_7_ | 540.2823 | 541.2896 | 12.47 | **P_7157** |
| TMC-1 D/ *Streptomyces* sp. A-230 |  |  |  |  |  |
| No microbial hits | C_85_H_119_N_7_O_32_  C_39_H_111_N_39_O_39_  C_98_H_220_N_24_O  C_113_H_224_N_12_  C_56_H_127_N_13_O_48_  C_54_H_115_N_27_O_38_  C_52_H_103_N_41_O_28_  C_68_H_103_N_33_O_23_  C_70_H_115_N_19_O_33_  C_72_H_127_N_5_O_43_ | 583.2633 | 584.2706 | 12.50 | **P_4884** |
| No microbial hits | C_94_H_166_N_44_O_19_  C_96_H_178_N_30_O_29_  C_98_H_190_N_16_O_39_  C_100_H_202_N_2_O_49_  C_157_H_299_NO  C_65_H_174_N_50_O_35_  C_113_H_194_N_4_O_38_  C_111_H_182_N_18_O_28_  C_81_H_174_N_42_O_30_  C_83_H_186_N_28_O_40_ | 738.4459 | 739.4531 | 12.57 | **P_3487** |
| No microbial hits | C_35_H_85_N_29_O_28_  C_33_H_73_N_43_O_18_  C_64_H_77_N_23_O_12_  C_66_H_89_N_9_O_22_  C_20_H_81_N_41_O_29_  C_52_H_101_N_3_O_37_  C_50_H_89_N_17_O_27_  C_48_H_77_N_31_O_17_  C_46_H_65_N_45_O_7_  C_49_H_73_N_35_O_13_ | 453.2040 | 454.2112 | 12.67 | **P_7024** |
